# Downstream Signaling Pathways in Mouse Adipose Tissues Following Acute In Vivo Administration of Fibroblast Growth Factor 21

**DOI:** 10.1371/journal.pone.0073011

**Published:** 2013-09-06

**Authors:** Eric S. Muise, Sandra Souza, An Chi, Yejun Tan, Xuemei Zhao, Franklin Liu, Qing Dallas-yang, Margaret Wu, Tim Sarr, Lan Zhu, Hongbo Guo, Zhihua Li, Wenyu Li, Weiwen Hu, Guoqiang Jiang, Cloud P. Paweletz, Ronald C. Hendrickson, John R. Thompson, James Mu, Joel P. Berger, Huseyin Mehmet

**Affiliations:** Discovery and Preclinical Sciences, Merck Research Laboratories, Merck Sharp & Dohme Corp., Whitehouse Station, New Jersey, United States of America; Boston University School of Medicine, United States of America

## Abstract

FGF21 is a novel secreted protein with robust anti-diabetic, anti-obesity, and anti-atherogenic activities in preclinical species. In the current study, we investigated the signal transduction pathways downstream of FGF21 following acute administration of the growth factor to mice. Focusing on adipose tissues, we identified FGF21-mediated downstream signaling events and target engagement biomarkers. Specifically, RNA profiling of adipose tissues and phosphoproteomic profiling of adipocytes, following FGF21 treatment revealed several specific changes in gene expression and post-translational modifications, specifically phosphorylation, in several relevant proteins. Affymetrix microarray analysis of white adipose tissues isolated from both C57BL/6 (fed either regular chow or HFD) and db/db mice identified over 150 robust potential RNA transcripts and over 50 potential secreted proteins that were changed greater than 1.5 fold by FGF21 acutely. Phosphoprofiling analysis identified over 130 phosphoproteins that were modulated greater than 1.5 fold by FGF21 in 3T3-L1 adipocytes. Bioinformatic analysis of the combined gene and phosphoprotein profiling data identified a number of known metabolic pathways such as glucose uptake, insulin receptor signaling, Erk/Mapk signaling cascades, and lipid metabolism. Moreover, a number of novel events with hitherto unknown links to FGF21 signaling were observed at both the transcription and protein phosphorylation levels following treatment. We conclude that such a combined "omics" approach can be used not only to identify robust biomarkers for novel therapeutics but can also enhance our understanding of downstream signaling pathways; in the example presented here, novel FGF21-mediated signaling events in adipose tissue have been revealed that warrant further investigation.

## Introduction

FGF21 is expressed in multiple metabolic tissues (liver, pancreas, and adipose, among other tissues [1]). Moreover, FGF21 increases glucose uptake and insulin sensitivity while decreasing gluconeogenesis, lipogenesis and circulating cholesterol levels in multiple pre-clinical animal models when administered pharmacologically [2] [3] [4] [5]. Fasting and ketogenic conditions induce hepatic expression of FGF21 through PPARα [6] [7], while its expression is induced in adipose tissues by PPARγ activation [8] [9]. FGF21 binds to fibroblast growth factor receptors (FGFRs) complexed with the co-receptor βKlotho (Klb) and signals through phosphorylation cascades involving FRS2a and Erk/MAPK leading to transcriptional changes of several metabolic genes [10] [11] [12]. Phosphorylation of Gsk-3, Shp2, Mek1/2, and Stat3 following FGF21 treatment of 3T3L1 adipocytes has been reported [13].

There is growing evidence suggesting that FGF21 signaling specifically in adipose tissue is crucial for its beneficial metabolic effects. For example, FGFR1 and βKlotho expression in adipose tissues, and downstream FGF21 signaling, are required for at least part of FGF21’s acute insulin-sensitizing and glucose uptake effects in mice [14,15]. In support of this observation, FGF21 displays impaired glucose- and triglyceride-lowering efficacy in lipodystrophic mice [16]. Recent reports have identified adiponectin as a key mediator of FGF21’s effects on glucose homeostasis and insulin sensitivity in mice [17,18]. In addition, administration of FGF21 has been shown to induce weight loss [3] [12] [19] and white adipose browning [20] in several pre-clinical animal models.

In order to further our understanding of FGF21 signaling downstream of receptor activation in mouse adipose tissues, we have used unbiased transcriptomic and phosphoproteomic profiling to quantify transcripts and phosphoproteins that are modulated after acute FGF21 treatment. For our studies, we used both native FGF21 protein (WT FGF21) and a pegylated form that increases its half-life [5] while displaying acute efficacy in terms of normalizing insulin-stimulated glucose uptake in insulin-resistant mice [21]. In addition to affecting known signaling cascades and metabolic pathways, FGF21 treatment also resulted in the phosphorylation and regulation of many novel proteins and transcripts with hitherto unknown links to FGF21 signal transduction pathways.

## Materials and Methods

### Ethics Statement

All animal procedures were reviewed and approved by the Institutional Animal Care and Use Committee of Merck & Co., Inc.

### In vivo studies for RNA profiling

Animals were housed in temperature-, humidity-, and light-controlled rooms (21–23°C, 47–65%, and 12/12-h light/dark cycle, respectively). Male C57BL/6 mice were purchased from Taconic Farms, Inc. at 6 weeks of age and fed either chow diet (Teklad 7012) or high fat diet (Research Diets D12492). Male db/db mice were purchased from The Jackson Laboratory at 5 weeks of age and fed chow diet (Teklad 5008). At 8 weeks of age, the mice were treated with either vehicle (saline), WT FGF21 or PEG30-FGF21 Q108 (Ambrx, San Diego, CA) [5] by subcutaneous injection twice daily (BID; WT FGF21 at 1.25 mg/kg) or twice weekly (BIW; PEG30-FGF21 Q108 at either 0.75 or 2.5 mg/kg) for either 2 or 5 days. This corresponded to 1 or 2 doses of PEG30-FGF21 Q108, respectively, with tissues taken approximately 30 hours post last dose. Tissues were taken approximately 6 hours post last dose for vehicle and WT FGF21. There were 5 animals per treatment group. Terminal ambient blood glucose levels were measured by glucometer (Onetouch Ultra). Mice were euthanized by CO2 asphyxiation 6 hours post last dose and adipose depots (epididymal, inguinal, and retroperitoneal white adipose tissues (EWAT, IWAT, and RPWAT) and brown adipose tissue (BAT)) were flash frozen in liquid nitrogen.

### RNA Extraction and Hybridization

Microarray analysis was performed as previously described [22]. Briefly, total RNA was isolated from frozen tissues after homogenizing in TRIzol reagent (Invitrogen, Carlsbad, CA) and processed with the Promega SV-96 total RNA kit (Promega, Madison, WI) according to the manufacturer’s instructions. Samples were amplified and labeled using a custom automated version of the NuGEN Ovation WB protocol. Hybridization on custom mouse Affymetrix microarrays (RM-MG01Aa520487, Mouse RSTA Custom Affymetrix 1.0. Santa Clara, CA.), labeling and scanning using Affymetrix ovens, fluidics stations and scanners were performed according to the protocols recommended (NuGEN, San Carlos, CA). Sample amplification, labeling, and microarray processing were performed by the Covance Genomics Laboratory in Seattle, WA. The raw gene expression data have been deposited in NCBI’s Gene Expression Omnibus database and are accessible at: http://www.ncbi.nlm.nih.gov/geo/query/acc.cgi?acc=GSE44095.

### Cell culture and in vitro treatments

3 T3-L1 preadipocytes (American Type Culture Collection) were grown in DMEM and differentiated as previously described [23]. The cells were used 9–11 days following differentiation induction, when exhibiting >90% adipocyte phenotype. Human adipocytes were obtained from Cell Applications, Inc. (San Diego, CA). Human pre-adipocytes were grown to confluence in pre-adipocytes medium (Cell Applications, Inc.). Differentiation was induced by incubating cells in adipocyte differentiation medium for 10 days. Differentiated 3T3-L1 and human adipocytes were serum depleted overnight in 0.5% fatty acid free BSA (Sigma–Aldrich, St. Louis, MO) and treated for 15 min with WT FGF21 or PEG30-FGF21 Q108 (1 μg/ml). After treatments, the cells were rinsed twice with PBS and scraped into ice-cold lysis buffer as described on Western blot methodology.

### In vitro studies for phosphoproteomic analysis

SILAC labeling for mouse 3T3L1 cells was performed in heavy (^13^C6 Lys and ^13^C6 Arg) and light (^12^C6 Lys and ^12^C6 Arg) DMEM media as previously described [24]. At passage five, the 3T3L1 cells were differentiated into adipocytes prior to treatment, as previously described [25]. The heavy or light Lys/Arg labeled cells were treated with vehicle or WT FGF21 at 1 μg/ml (Ambrx, San Diego, CA) for 10 min. After the treatment, the cells were lysed and the protein concentrations of lysates were measured in five replicates using BCA protein assays [Thermofisher Scientific, Rockford, IL]. Equal amounts of vehicle or FGF21 treated light and heavy stable isotope labeled lysates were mixed to generate the forward and reverse labeling samples to minimize potential labeling discrepancies. Aliquots of the sample (~500 μg) were subjected to proteolytic digestion with Trypsin and Endo-Protease LysC prior to further sample processes using Phos-Trap ^TM^ phosphopeptide enrichment kit (PerkinElmer, Waltham, MA) following the manufacturer’s instructions. All samples were analyzed using a NanoAquity (Waters Corp., Milford, MA) coupled with a LTQ-Orbitrap (Thermo-Fisher Scientific, Waltham, MA). In each data collection cycle, a high resolution full MS spectrum (300–1500 m/*z*) was acquired in the Orbitrap (6 × 10^4^ resolution setting, automatic gain control (AGC) target of 10^6^) followed by 10 tandem mass spectra were acquired in the LTQ using collision-induced dissociation (CID) (AGC target, 10000; threshold 5000) in a data dependent fashion (dynamically exclusion, 30 s). Raw full MS data were analyzed by Elucidator SILAC analysis tool (Microsoft, Redmond, WA, version 3.3) to determine relative ratios of "light" to "heavy" peptide pairs. MS/MS data were then searched against the Merck rodent database using the SEQUEST algorithm. The resulting phosphopeptides that exhibited >1.5 fold changes in both "forward" and "reverse" conditions were verified by manual interpretation and were included in the Table S9.

### In vivo validation study

Male C57BL/6 mice were purchased from Taconic Farms, Inc. at 12 weeks of age and fed chow diet (Teklad 7012). At 14 weeks of age, the mice were given a single subcutaneous injection of either vehicle (saline) or PEG30-FGF21 Q108 at one of three doses (2.5, 0.75, or 0.25 mg/kg). There were 8 animals per group. For phosphoprotein analysis, tissues were taken 15 min post injection and flash frozen. For RNA quantitation by PCR analysis and serum immunoassay analysis, IWAT and terminal blood were taken 6 hr, 24 hr, or 48 h post injection. Insulin was measured using mouse ultrasensitive insulin ELISA kit (Mercodia Inc, Wiston Salem, NC).

### Quantitative Real-Time PCR

For real-time PCR analysis, total RNA was isolated from frozen iWAT tissues by tissue homogenization with disposable probes and RNA extraction using the Qiagen RNeasy Lipid Tissue Mini Kit (Qiagen, Valencia, CA). RNAs were treated in-solution with Turbo DNase in the presence of SUPERase-In (both Life Technologies, Grand Island, NY), followed by purification and concentration using the Qiagen RNeasy MinElute Cleanup Kit. Purified and DNase-treated RNAs were quantified by absorbance at 260 nM. 600 ng of RNA was converted to cDNA using the RT2 PCR Array First Strand Kit (SABiosciences, Valencia, CA). Custom-made PCR arrays (CAPM-0838E; SABiosciences) containing pre-developed reactions for 24 customized genes, four housekeeping genes, and four control reactions were performed according to the manufacturer’s instructions. Data analysis was performed using the web-based analysis tool (www.sabiosciences.com/pcrarraydataanalysis.php). Relative gene expression was calculated by 2^-^ *ΔΔ* ^Ct^ relative to control. Treatment effects were evaluated by independent T tests of group means using Bonferroni adjustments for multiple comparisons. Statistical significance was defined as p < 0.05

### Western blotting

Frozen tissues were homogenized on ice in Cell Lysis Buffer (Cell Signaling Technology, Danvers, MA) supplemented with protease and phosphatase inhibitors

(Sigma–Aldrich, St. Loius, MO.) Lysates were sonicated for 15 sec. and centrifuged at 14,000g for 15 min at 4 C. The infranatant was collected below the supernatant fat cake. Protein concentration was determined by the bicinchoninic acid method (Thermo Fischer-Pierce Inc, Rockford, IL). 40 μg of each lysate was resolved in 4–20% Tris–glycine SDS–PAGE, transferred to nitrocellulose membranes and blotted with antibodies (Cell Signaling Technology, Danvers, MA) against phospho Frs2α, phospho Erk1/2, phospho Mypt1, phospho Mdm2, phospho Stat3, phospho Cbx5 and β-actin, which served as a loading control. Chemiluminescence (Super Signal West Femto, Pierce Biotechnology, Inc., Rockford, IL) was quantified by laser densitometry using FluorChem HD2 chemiluminescence system (Proteinsimple Inc, Santa Clara, CA) within the linear range of detection.

### Immunoassays

After 24 and 48 h of treatment, blood was obtained from overnight-fasted mice by terminal exsanguination. Plasma Igf1 was measured by Quantikine immunoassay from R & D Systems Inc (Minneapolis, MN). Plasma Ccl11, Cxcl2, Il1rn and Kitl levels were measured by sandwich immunoassays developed using the commercially available DuoSet ELISA Development kits DY420, DY452, DY480, and DY455, respectively, from R & D Systems, Inc (Minneapolis, MN). Briefly, antibody against each protein was captured on assay plate by incubating with antibody solution (0.8 µg/ml in PBS, 100 μl per well) overnight in 96-well plates at 4^0^C followed by blocking with 200 µl per well of 1% BSA in PBS for 1 h at RT. 100 µl of plasma was added per well and incubated for 2 h at RT, followed by incubation for 1 h with biotinylated detection antibody (0.4 µg/ml, 100 µl per well). 100 µl per well of Eu (Europium)-Streptavin (#1244-360, PerkinElmer, Waltham, MA) was added and incubated for 0.5 hr, followed by adding 100 µl per well of Enhancement solution (#1244-105, PerkinElmer). Plates were washed with 200 µl per well PBS containing 0.05% tween 20 and fluorescence measured with an Envision plate reader. The concentration of each protein in plasma was extrapolated from a standard curve generated in the same assay using recombinant protein that was included in the DuoSet ELISA Development kit.

### Statistical Analysis

Gene expression data were processed using the RMA pipeline in the Rosetta Resolver software (Microsoft, Redmond, WA) to obtain the MAS5 p values. One-way and N-way ANOVA analyses were performed with Matlab (The Mathworks, Natick, MA). For 1-way ANOVA analysis, probe sets had to pass a pre-filter of Affymetrix MAS5 present call p-value < 0.05 in > 50% of the samples to qualify for further analysis. Average fold changes between compound and vehicle treatment groups were generated with the vehicle as baseline. Differentially expressed probe sets (signature genes) were selected by applying 1.2 fold change (up- and down-regulated) and 1-way ANOVA p value < 0.05. N-way ANOVA analysis was performed by incorporating all FGF21 treatments for 2 days and 5 days and mouse stains/disease models (db/db, WT chow and WT HFD) by using the following fitting equation: GeneExpression = C + α* FGF21 + β* Animal + γ* Day + Interaction terms + ε. The resulting N-way ANOVA p values were used in down-stream filtering as described. Monte Carlo simulations were carried out to estimate the false discovery rate for the gene selection. Rosetta Resolver was used to generate the heatmaps, Matlab was used to generate the log intensity box plots, and Tibco Spotfire 4 (Tibco Spotfire, Somerville, MA) was used to generate the bar graphs and the scatter plot. The Ingenuity Pathway Analysis tool (Ingenuity Systems, Redwood City, CA) was used to analyze the enrichment of biological functions in the signature genes, while the Gene Set Annotator in the Merck, Target and Gene Information System was used to identify enriched GO terms. The p values were calculated by using the hypergeometric distribution and the E values from the Bonferroni-adjusted p values.

All other results are expressed as the mean ± S.E.M. The data were analyzed by Student’s t-Test and graphed using Tibco Spotfire 4 (Tibco Spotfire, Somerville, MA). P values less than 0.05 were considered statistically significant.

## Results

### Gene Expression Changes in White Adipose Tissues (WAT)

**Figure 1:**
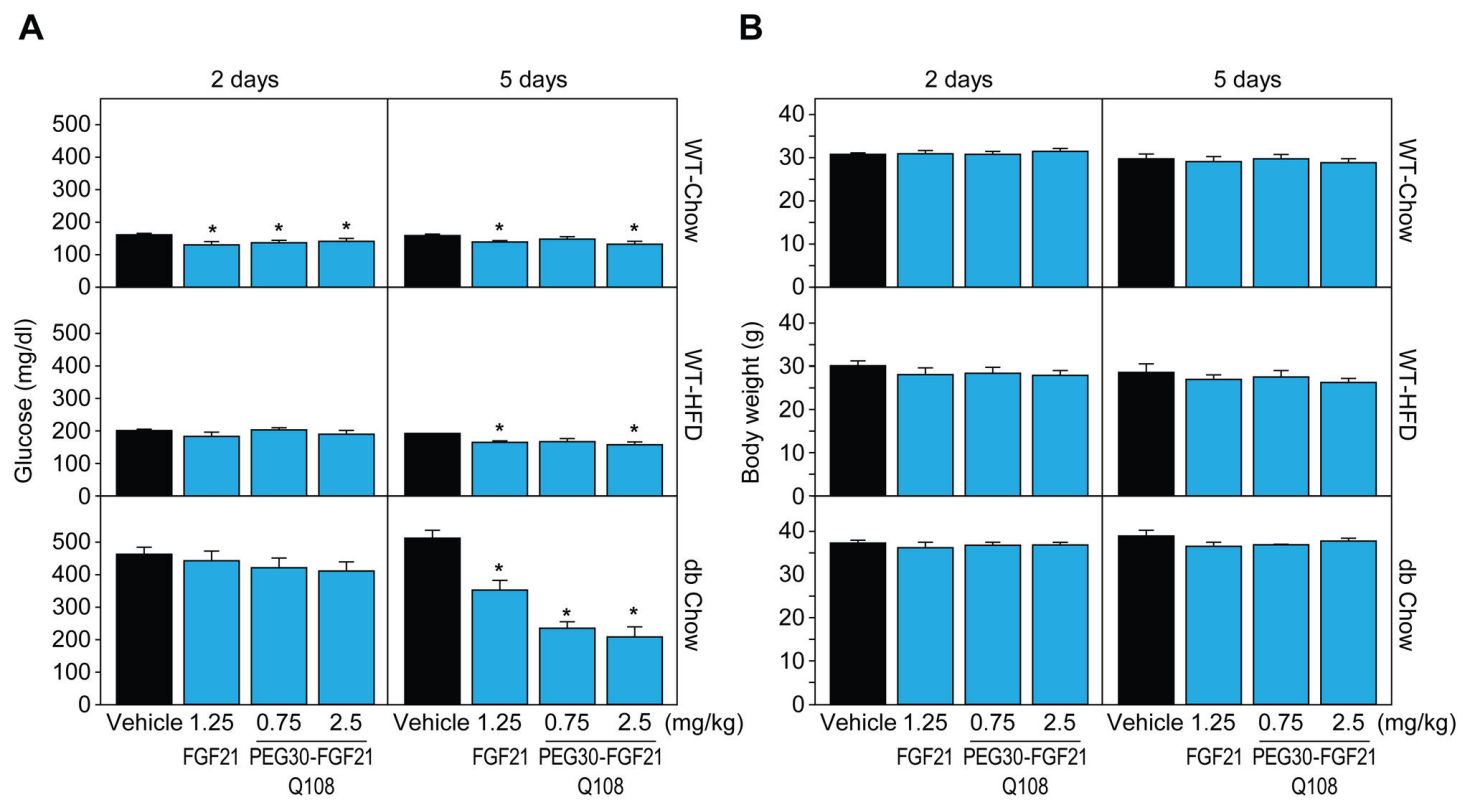
FGF21 treatment lowers plasma glucose levels while not affecting body weights. Glucose (A) and body weight (B) measurements are plotted for each mouse model (WT Chow, WT HFD, and dbdb Chow) across each treatment (WT FGF21 and 2 doses of PEG30-FGF21 Q108). Error bars denote standard error. Asterisks denote significant change compared to vehicle treatment within each mouse model and time point (students t-test p value < 0.05).

To identify genes robustly regulated by FGF21 acutely in white adipose tissues (WAT) we used 3 WAT depots (IWAT, EWAT, and RPWAT) and three mouse models (WT-chow, WT-HFD, and db/db-chow) treated with FGF21 either acutely or sub-chronically. Terminal ambient blood glucose levels were significantly decreased in all three mouse models by FGF21 treatment (from 159 to 135 mg/dl (WT-chow), 186 to 159 mg/dl (WT-HFD), and 512 to 212 mg/dl (db/db-chow), representing vehicle and PEG30-FGF21 Q108 2.5 mg/kg treatments respectively at the 5 day time point, (all p<0.05); Figure 1). Body weights remained unchanged over the 5 day period. Plasma TG levels were not measured but we have previously found that these are decreased under these conditions after 12 days of treatment [5].

Overall responses, in terms of the number of probe sets meeting the 1.2 fold and p<0.05 cutoff, were comparable between the WAT depots although the responses were slightly stronger at the 5 day time point (compared to the 2 day time point), possibly due to increased secondary effects with sub-chronic dosing (see Figure S1). N-way ANOVA analysis was performed to identify consistent gene expression signatures across a range of time points, treatment paradigms, and mouse model for each independent WAT depot. The averaged numbers of the false positive genes were obtained from 500 times of the Monte Carlo simulation results, and the false discovery rates for the N-way ANOVA analysis under the stringent cut of p<0.001 was estimated to be at 1~2% level for all three WAT depots. 1129 probe sets were identified that had an N-way ANOVA p<0.001 between FGF21 and vehicle treatments in the three WAT depots (intersection, see Table S1).

GO term pathway enrichment analysis revealed that genes involved in multiple metabolic processes, cell death, and signaling were significantly represented in this data set (Table S2). Similarly, the Ingenuity Pathway Analysis tool was used to identify canonical pathways that were enriched, which included UDP-N-acetyl-D-glucosamine Biosynthesis II, p53 Signaling, and Triacylglycerol Biosynthesis (Table S3). The genes associated with each GO term or canonical pathways are listed in Tables S2 and S3 while the gene expression data for a subset of the canonical pathways are shown in Table S4. These included FGF signaling (Dusp4 and Spry4 were up-regulated, while Itpr1, Pik3r3, and Map2k6 were down-regulated following FGF21 treatment), Wnt/β-catenin signaling (Hdac1 and Ppp2r1b were up-regulated while Jun, Dkk2, and Sfrp5 were down-regulated), glucose uptake (Insr, Slc1a3 (Glut1) and Stx6 were up-regulated), and amino acid transport (slc6a9, Slc6a8, and Slc6a13 were down-regulated). Four genes in this signature were previously reported to be regulated by FGF21 in WAT (PPARγ, Dgat1, and Ucp1 were up-regulated, while Ucp3 was down-regulated [12]). Some genes only reached maximal expression after sub-chronic treatment (5 day time point), such as Ucp1 (12 fold up-regulation in IWAT) and Slc1a3 (Glut1; 2 fold up-regulation in RPWAT).

**Figure 2:**
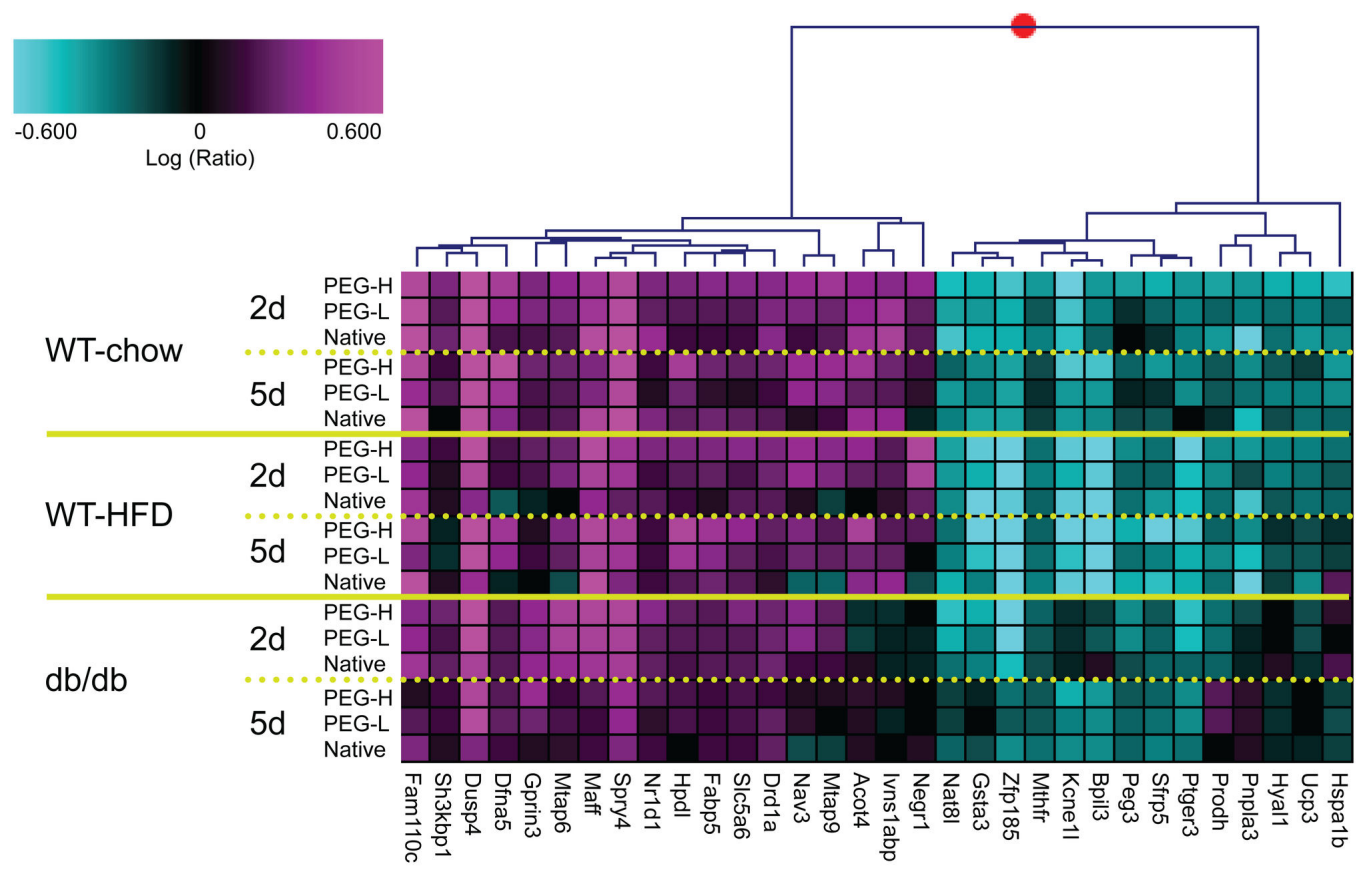
Acute FGF21 treatment-induced RNA markers in white adipose tissues. The top 32 RNA markers (Table 1) from IWAT are represented in the clustergram (see Figure S2 for EWAT and RPWAT). Plotted are the logRatio values on a scale of +/- 0.6 (+/- 4 fold) with magenta and cyan signifying up- and down-regulated genes, respectively. Each row is the average of up to 5 animals for that treatment group. Gene names are shown below the clustergram. Native = WT FGF21; PEG-L and PEG-H = PEG30-FGF21 Q108 at 0.75 and 2.5 mg/kg, respectively; and 2d and 5d correspond to number of days of treatment.

Of the 1129 probe sets, 165 were also significantly regulated at the high dose of PEG30-FGF21 Q108 (2.5 mg/kg) acutely (2 day) in IWAT of WT-chow fed mice (at least 1.5 fold change and 1-way ANOVA p<0.05 compared to vehicle treatment, Table S1). The top 32 up- and down-regulated probe sets are plotted in Figure 2 and Figure S2, and are listed in Table 1, across the three white adipose depots. Genes involved in feedback regulation of Fgfr signaling (Dusp4 [26] and Spry4 [27]) were among the most robustly induced genes acutely, while the expression of several genes involved in lipid metabolism were either increased (Acot4 [28] and Fabp5 [29]) or decreased (Pnpla3 [30] and Ucp3 [31]). Of note, a few of these genes may be clinically relevant since they showed opposite regulation between A) FGF21 treatment (vs vehicle treatment), and B) the “disease” phenotype (HFD vs chow, or db/db vs WT). For example Negr1 was down-regulated in “disease” but up-regulated by FGF21 treatment while Sfrp5 was up-regulated by “disease” and down-regulated by FGF21 treatment (see Table S1 and Figure S3).

**Table 1:** Acute FGF21 treatment-induced gene expression changes in inguinal white adipose tissue (IWAT) from WT mice on chow diet.

Probeset ID	Gene Symbol	Entrez Gene ID	WT Chow. PEG30-FGF21 Q108, 2.5 mg/kg, 2d / Vehicle Treatment						Validation Study. IWAT. Fold Change		
			IWAT		EWAT		RPWAT		n=5	n=5	n=5
			Fold Change	ANOVA p	Fold Change	ANOVA p	Fold Change	ANOVA p	0.25 mg/kg	0.75 mg/kg	2.5 mg/kg
merck-AK012530_at	Dusp4	319520	6.6	0.000	5.6	0.000	4.1	0.000	3	1.8	1.5
merck-NM_011898_at	Spry4	24066	3.3	0.001	3.0	0.000	2.9	0.000	4.8	2.7	2.3
merck-NM_027828_at	Fam110c	104943	3.3	0.000	2.3	0.000	2.2	0.000	11.3	5.8	9.8
merck-NM_018769_at	Dfna5	54722	2.6	0.000	1.8	0.003	1.6	0.021	7.8	4.6	5.5
merck-NM_010755_at	Maff	17133	2.4	0.001	3.1	0.000	2.0	0.009	5.3	3.1	2.9
merck-AK147287_at	Mtap9	213582	2.3	0.015	1.8	0.001	1.6	0.013	5.8	2.4	2.6
merck-NM_134247_at	Acot4	171282	2.2	0.006	1.9	0.017	1.5	0.120	1.8	1.1	1.6
merck-AK053042_at	Nav3	260315	2.2	0.008	2.2	0.000	2.2	0.000	3.9	2	3.1
merck-NM_177274_at	Negr1	320840	2.1	0.001	1.6	0.054	1.5	0.039	3.5	1.3	2
merck-BC053039_at	Mtap6	17760	2.1	0.001	2.4	0.001	2.1	0.013	2.9	1.4	1.9
merck-NM_001039511_s_at	Ivns1abp	117198	2.1	0.003	1.8	0.001	1.4	0.112	3.3	1.3	1.9
merck-NM_183183_at	Gprin3	243385	1.9	0.006	1.2	0.185	1.1	0.366			
merck-NM_177870_at	Slc5a6	330064	1.9	0.000	1.6	0.099	1.4	0.082			
merck-NM_010634_at	Fabp5	16592	1.9	0.011	2.1	0.001	1.8	0.005	5.2	2.5	3.6
merck-NM_010076_at	Drd1a	13488	1.9	0.001	1.8	0.002	1.5	0.003			
merck-NM_145434_at	Nr1d1	217166	1.9	0.001	1.9	0.002	1.6	0.026			
merck-AK028299_at	Sh3kbp1	58194	1.9	0.001	1.6	0.042	1.6	0.045			
merck-NM_146256_at	Hpdl	242642	1.8	0.016	1.8	0.000	2.1	0.001			
merck-NM_010840_at	Mthfr	17769	-2.0	0.001	-1.8	0.000	-2.0	0.000			
merck-NM_054088_at	Pnpla3	116939	-2.1	0.003	-1.7	0.018	-1.8	0.000			
merck-NM_011196_at	Ptger3	19218	-2.1	0.038	-2.1	0.012	-2.0	0.004			
merck-NM_011172_at	Prodh	19125	-2.2	0.000	-1.8	0.003	-1.6	0.001	-2.5	-4.8	-4.7
merck-NM_199303_at	Bpil3	228796	-2.2	0.019	-2.1	0.002	-1.9	0.023			
merck-BP753991_at	Peg3	18616	-2.2	0.000	-2.5	0.001	-1.8	0.001	-1.8	-3.5	-2.9
merck-NM_008317_at	Hyal1	15586	-2.3	0.000	-1.6	0.001	-1.7	0.000	-5.2	-4.7	-5.8
merck-NM_010356_s_at	Gsta3	14859	-2.3	0.006	-1.4	0.013	-1.5	0.006	-1.2	-1.6	-1.2
merck-NM_009464_at	Ucp3	22229	-2.3	0.001	-3.3	0.000	-2.2	0.000	-1.8	-2.3	-1.7
merck-NM_001001985_at	Nat8l	269642	-2.4	0.001	-2.8	0.011	-2.6	0.000	1.6	-2	1.1
merck-NM_018780_at	Sfrp5	54612	-2.5	0.017	-4.4	0.017	-1.6	0.035	-2	-1.3	-3.7
merck-AK159139_at	Hspa1b	15511	-2.6	0.004	-1.5	0.001	-5.6	0.001	-2.2	-2.8	-2.7
merck-NM_009549_at	Zfp185	22673	-3.0	0.000	-1.6	0.003	-2.2	0.000	2.6	1	1.4
merck-NM_021487_at	Kcne1l	66240	-3.6	0.001	-3.0	0.001	-2.8	0.000	-15.6	-43	-31.6

Listed are the top 32 probe sets, out of 165, that were regulated in IWAT by a single 2.5 mg/kg dose of PEG30-FGF21 Q108 (1.5 fold and p<0.05). The fold change and 1-way ANOVA p values for the acute treatment of PEG30-FGF21 Q108 (2.5 mg/kg) in EWAT and RPWAT are included as is the data from the validation study (qPCR). The 1129 probe sets, from which the 165 were derived, are listed in Table S1.

**Figure 3:**
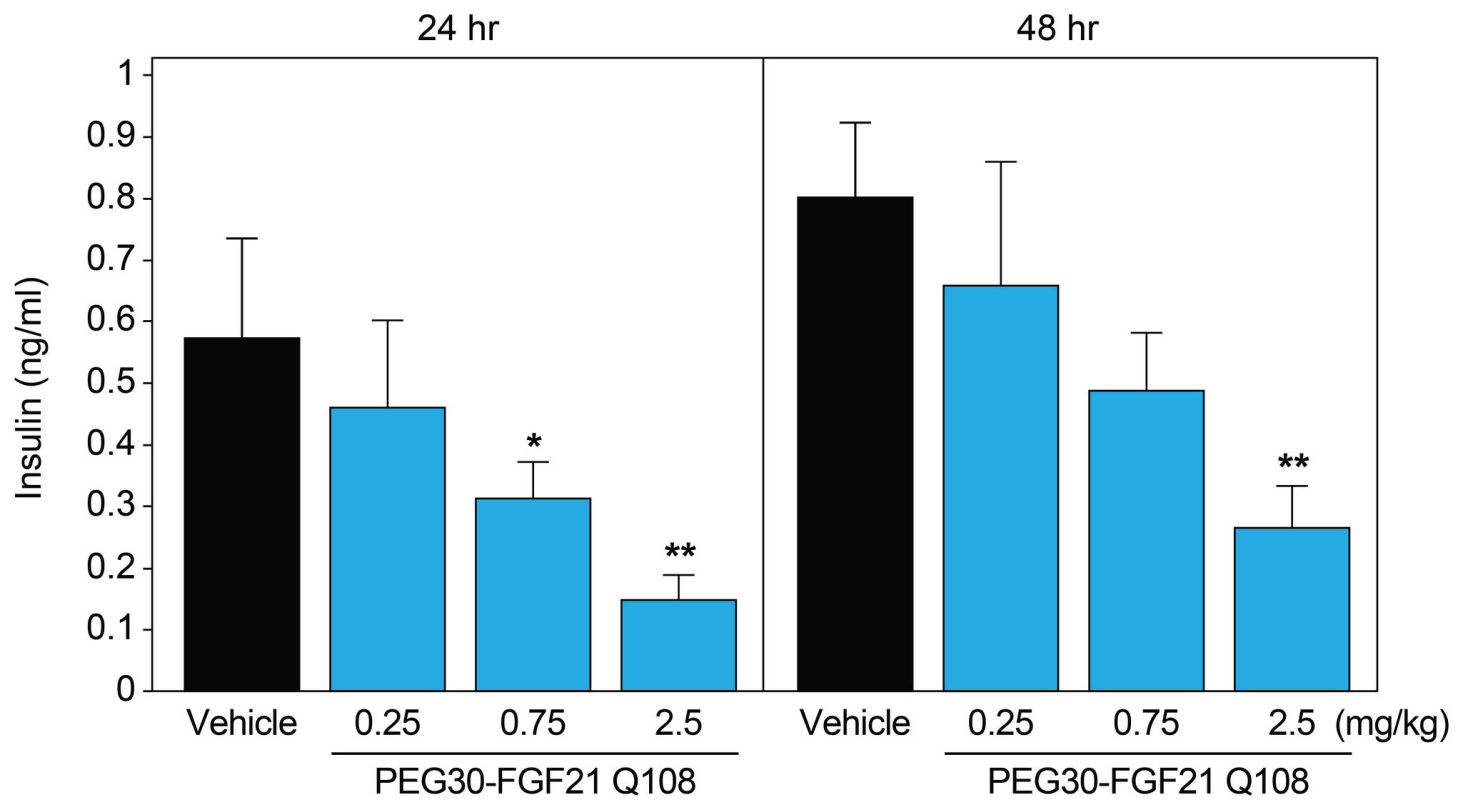
Acute FGF21 treatment lowers plasma insulin levels. C57BL/6 mice on chow diet were treated with a single dose of vehicle or PEG30-FGF21 Q108 at one of three doses (0.25, 0.75, or 2.5 mg/kg). Plasma insulin levels were measured 24 and 48 hours post dose. Error bars denote standard error. Asterisks denote significant change compared to vehicle treatment within each time point (students t-test p value < 0.05*, p<0.01**).

In an independent *in vivo* validation study, C57BL/6 mice (on chow diet) were treated with a single injection of PEG30-FGF21 Q108 at doses ranging from 0.25 to 2.5 mg/kg and adipose tissues flash frozen at 15 min, 24 hours, and 48 hours post-dose. Blood was collected at the 24 h and 48 h time points. Insulin levels were decreased in a dose-dependent manner at the 24 h and 48 h time points (from 0.57 to 0.15 ng/ml and from 0.8 to 0.27 ng/ml for the 24 and 48 h time points, respectively; Figure 3). Quantitative PCR analysis of 22 of the top 32 genes was performed using RNA from IWAT samples at the 24 h time point with 20 of the 22 genes showing regulation by FGF21 in agreement with the microarray study (Table 1). Interestingly, even the lowest dose of PEG30-FGF21 Q108 (0.25 mg/kg) in the validation study showed robust regulation of these genes in IWAT, highlighting the sensitivity of these markers. Perhaps, this is not surprising since PEG30-FGF21 Q108 at this dose (0.25 mg/kg) was recently reported to have beneficial effects in terms of normalizing insulin-stimulated glucose uptake in a diet-induced mouse model of insulin-resistance after acute treatment [21].

### FGF21 Transcriptional Effects in Brown Adipose Tissue (BAT)

**Figure 4:**
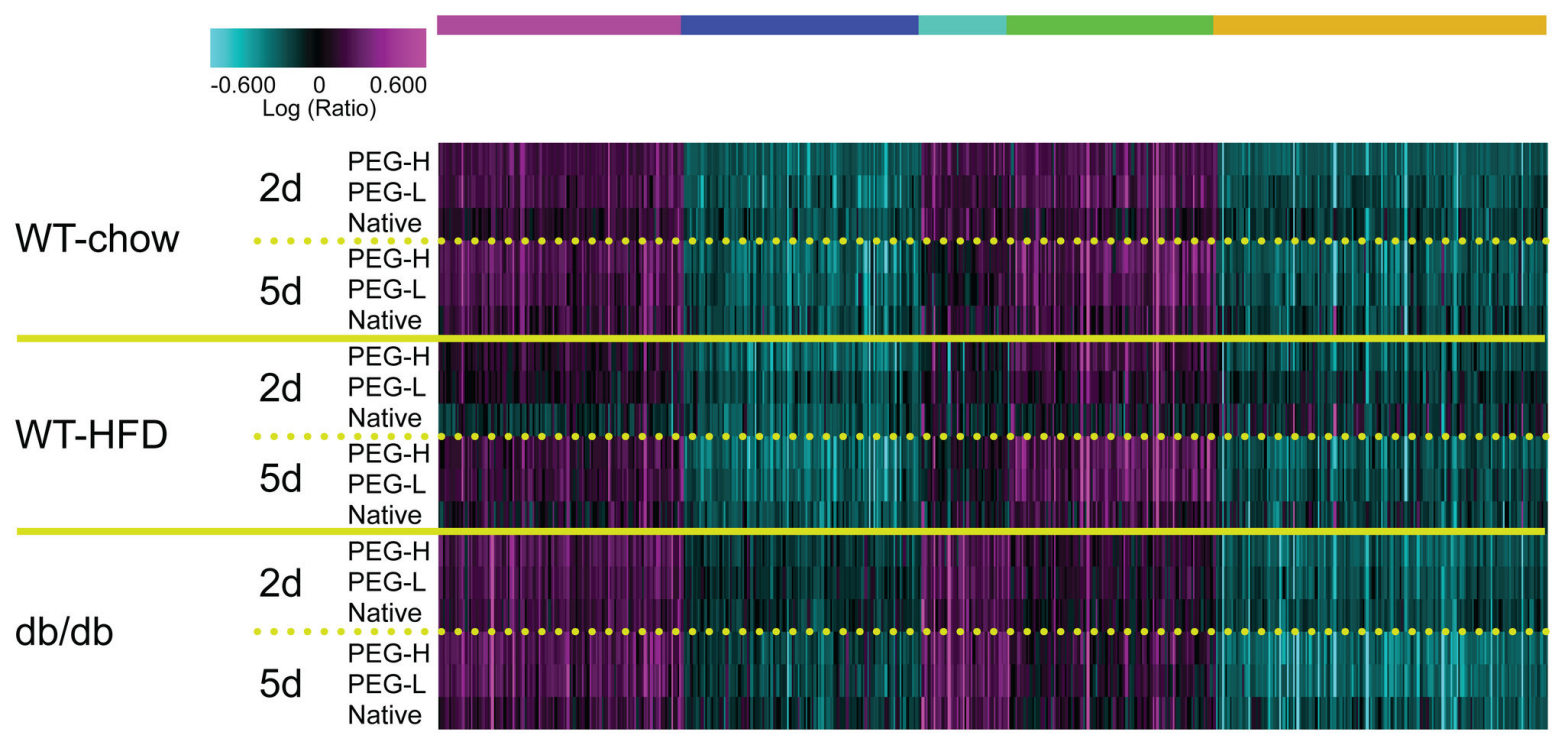
Acute FGF21 treatment induces robust and consistent changes in brown adipose tissue gene expression. Shown in the clustergram are the 1634 probe sets regulated by a single 2.5mg/kg dose of PEG-30-FGF21 Q108 in WT mice on chow diet. Clustergram properties are the same as in Figure 2. The 1634 probe sets are listed in Table S5.

RNA from BAT was also analyzed by Affymetrix microarrays following FGF21 treatment. Of the adipose tissues profiled, FGF21 treatment had the most robust effect in BAT in terms of the number of probe sets that were statistically significantly changed compared to vehicle treatment, especially at the 5 day time point (see Figure S1). N-way ANOVA analysis was performed using all the doses of the WT and PEG30-FGF21 Q108 in all three mouse models (WT-chow, WT-HFD, and db/db-chow). Approximately 8500 probe sets had N-way ANOVA p<0.001 of which 1634 were also significantly regulated by the high dose of the PEG30-FGF21 Q108 (2.5 mg/kg) acutely (2 day) in BAT of WT-chow fed mice (1.2 fold change and 1-way ANOVA p<0.05 compared to vehicle treatment (Figure 4 and Table S5). Enriched canonical pathways from the Ingenuity Pathway Analysis tool included Phosphatidylglycerol and Triacylglycerol Biosynthesis, Erk/Mapk signaling, and Insulin Receptor Signaling (see Table S6). Robustly regulated genes included Sgk2, Dusp4, Spry4 and Lcn2 (up-regulated) and Hspa1a, Hspa1b, Nr4a1, Nr4a3, and FosB (down-regulated). Interestingly, the expression levels of the majority of the top 32 FGF21-regulated genes identified in WAT after treatment with FGF21 approached the levels observed in BAT (either up- or down-regulated), in support of a white adipose “browning” effect by this treatment (see Figure S4) [20]). Examples included Dusp4, Kcne1l, and Sfrp5 (Ucp1 was included in Figure S4 as a “browning” control).

### Blood Borne Protein validation of Gene Expression Signatures in White Adipose Tissues

More than 6500 probe sets were interrogated in WAT, of which approximately 2500 were significantly regulated by the high dose of the PEG30-FGF21 Q108 (2.5 mg/kg) acutely (2 day) in at least one of the three WAT tissues (1.2 fold change and 1-way ANOVA p<0.05) compared to the corresponding vehicle treatment. Detailed bioinformatics analysis led to the identification of transcripts, from white adipose tissues, which encoded secreted proteins. From the (differentially regulated) 2500 probe sets, 334 genes were annotated as secreted proteins (Swissprot keywords or extracellular localization, Table S7). The top 20 up- and down-regulated probe sets are listed in Table 2. Il1rn was added to this list even though the 1-way ANOVA p values for IWAT and EWAT were above 0.05 (they were 0.07 and 0.08, respectively).

**Table 2:** FGF21 treatment-induced gene expression changes of adipose tissue transcripts encoding potential secreted proteins.

Probeset ID	Gene Symbol	Entrez Gene ID	WT Chow. PEG30-FGF21 Q108, 2.5 mg/kg, 2d / Vehicle Treatment						Validation Study. IWAT. Fold Change		
			IWAT		EWAT		RPWAT		n=5	n=5	n=5
			Fold Change	ANOVA p	Fold Change	ANOVA p	Fold Change	ANOVA p	0.25 mg/kg	0.75 mg/kg	2.5 mg/kg
merck-AK053042_at	Nav3	260315	2.2	0.008	2.2	0.000	2.2	0.000	3.9	2	3.1
merck-NM_177274_at	Negr1	320840	2.1	0.001	1.6	0.054	1.5	0.039	3.5	1.3	2
merck-NM_001024698_at	Cpa2	232680	2.0	0.225	2.3	0.016	3.7	0.026			
merck-NM_010171_at	F3	14066	1.7	0.023	1.7	0.005	1.9	0.001			
merck-AF128205_a_at	Ccl11	20292	1.6	0.086	1.0	0.986	1.6	0.020			
merck-NM_010512_at	Igf1	16000	-1.1	0.035	-1.2	0.036	-1.2	0.017			
merck-NM_010517_s_at	Igfbp4	16010	-1.3	0.000	-1.3	0.000	-1.2	0.004			
merck-NM_008520_at	Ltbp3	16998	-1.4	0.008	-1.4	0.000	-1.5	0.000			
merck-NM_008489_at	Lbp	16803	-1.4	0.001	-1.5	0.003	-1.3	0.116			
merck-ENSMUST00000015791_at	Lama5	16776	-1.5	0.001	-1.2	0.038	-1.3	0.019			
merck-NM_007925_at	Eln	13717	-1.5	0.003	-1.1	0.087	-1.2	0.083			
merck-BB213274_s_at	Kitl	17311	-1.7	0.001	-1.2	0.036	-1.4	0.007			
merck-NM_030565_at	Fam20c	80752	-1.7	0.001	-2.1	0.000	-2.0	0.000			
merck-ENSMUST00000063029_at	Chrdl1	83453	-1.7	0.010	-1.6	0.043	-1.4	0.069			
merck-NM_172874_at	Podn	242608	-1.8	0.002	-1.5	0.045	-1.7	0.002			
merck-AK190654_a_at	C2	12263	-1.8	0.005	-1.5	0.036	-1.6	0.010			
merck-NM_008317_at	Hyal1	15586	-2.3	0.000	-1.6	0.001	-1.7	0.000	-5.2	-4.7	-5.8
merck-NM_001039701_a_at	Il1rn	16181	-2.3	0.069	-1.7	0.080	-1.2	0.528			
merck-NM_018780_at	Sfrp5	54612	-2.5	0.017	-4.4	0.017	-1.6	0.035	-0.5	-0.8	-3.7
merck-NM_011704_at	Vnn1	22361	-2.6	0.000	-1.8	0.061	-1.8	0.011			
merck-NM_009140_at	Cxcl2	20310	-4.7	0.003	-1.6	0.044	-2.0	0.042			

Listed are the top 20 potential secreted proteins (in addition to IL1rn), out of 334, that were regulated by a single 2.5 mg/kg dose of PEG30-FGF21 Q108 (1.2 fold and p<0.05) in at least one of the three white adipose tissues (IWAT, EWAT, RPWAT). The 334 probe sets, from which the 20 were derived, are listed in Table S7.

**Figure 5:**
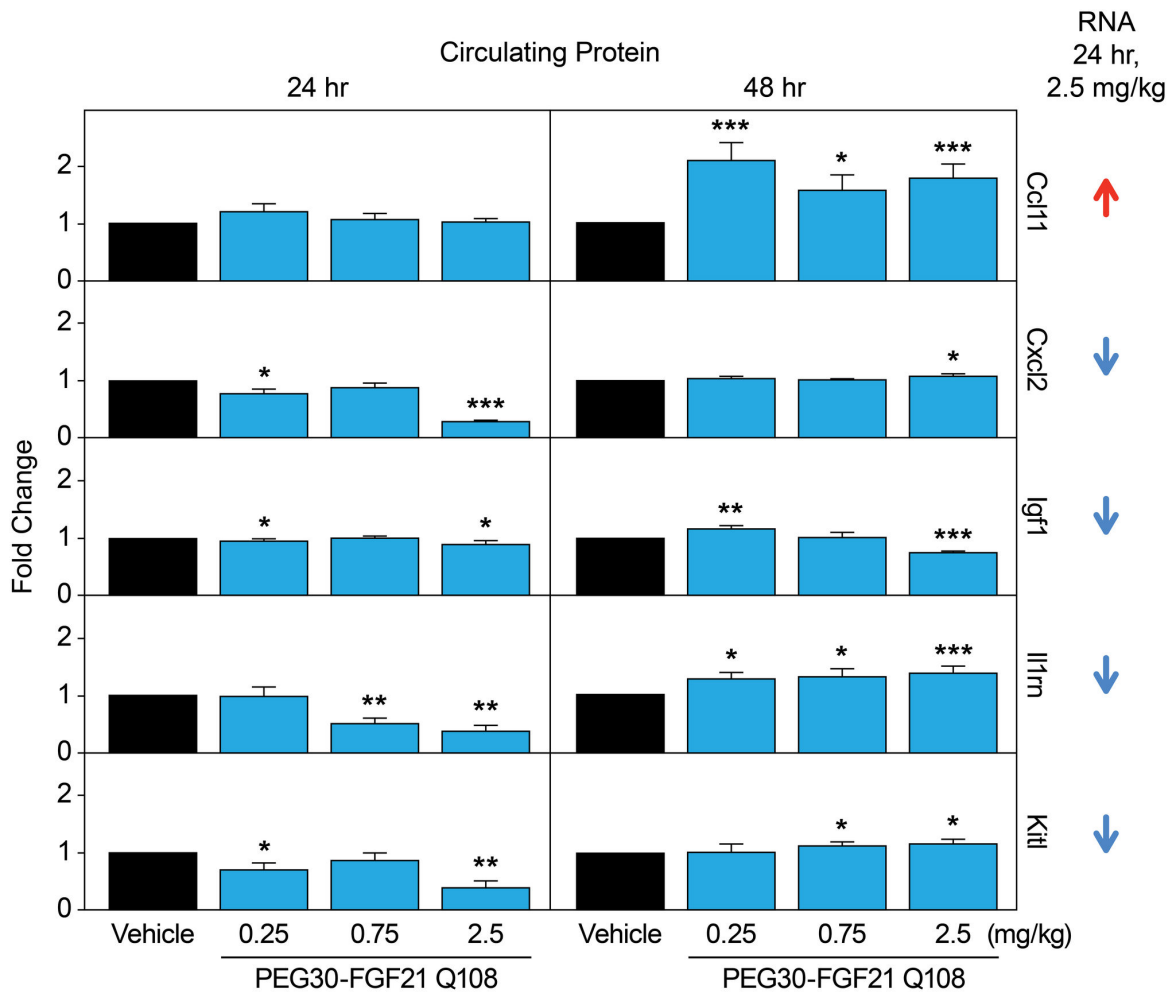
Plasma levels of secreted proteins identified in WAT by Affymetrix microarray profiling. C57BL/6 mice on chow diet were treated with a single dose of vehicle or PEG30-FGF21 Q108 at one of three doses (0.25, 0.75, or 2.5 mg/kg). Plasma Ccl11, Cxcl2, Igf1, Il1rn, and Kitl levels were measured 24 hours and 48 hours post dose (Table S8). Error bars denote standard error. Asterisks denote significant change compared to vehicle treatment within each time point (students t-test p value < 0.05*, p<0.01**, p<0.001***). Arrows on far right denote effects of PEG-FGF21 Q108 at 2.5 mg/kg at the RNA level after 24 hours of treatment (2 day time point) in WT chow-fed mice (IWAT).

In an independent *in vivo* validation study, immunoassays were performed using commercially available antibodies against 5 proteins and mouse blood taken at the 24 h and 48 h time points. Consistent with the gene expression data, Ccl11 protein levels were increased (at the 48 h post dose, Figure 5 and Table S8). The lack of increase in *circulating* Ccl11 at 24 hours post FGF21 treatment may reflect the specific differences in the kinetics of protein synthesis and/or secretion. Il1rn protein levels were significantly decreased at the 24 h time point following the high dose of PEG30-FGF21 Q108 (Figure 5). This is in agreement with the microarray analysis where the Il1rn transcript was down-regulated by the 2.5 mg/kg dose of PEG30-FGF21 Q108 at the 2 day time point. The increase in Il1rn at 48 h post-dose may reflect feedback regulation, although this requires further experimentation. Immunoassays for the three other proteins (Cxcl2, Igf1, and Kitl) did not show dose-dependent changes in the plasma after FGF21 treatment (Figure 5). There was, however, a significant decrease in the plasma levels of all three proteins at the highest dose of FGF21 tested at the 24 h time point in agreement with their transcript regulation. Further studies are required to fully validate these findings.

In the context of secreted proteins, the insulin-sensitizing adipokine adiponectin is of particular relevance to FGF21 signaling. Two recent studies [17,18] have shown that adiponectin is a downstream effector of FGF21. Furthermore, treatment with FGF21 enhanced both the expression and secretion of adiponectin in mouse adipocytes (differentiated *in vitro*), and also increased the serum levels of adiponectin in mice. In the present study we did not see significant increases in adiponectin mRNA in adipose tissues *in vivo*. In parallel experiments, we treated 3T3L1 adipocytes with WT FGF21 at 0.3 and 1 μg/ml and could not observe an increase in adiponectin protein in the media, despite increased Erk phosporylation, a robust indicator of FGF21 receptor activation (Figure S5A). Moreover, rosiglitazone (10 μM) treatment did trigger a 65% increase in secreted adiponectin in the same experiment. Similarly, in mice treated with WT FGF21 (at 1 mg/kg) a 2-fold increase in Erk phosphorylation, indicative of target engagement (after 15 minutes of treatment), was not associated with increased levels of plasma adiponectin after either 1 or 6 hours of treatment (Figure S5B). Rosiglitazone was not used in this *in vivo* study since more chronic dosing has been reported to be required to observe an increase in circulating adiponectin levels [32]. Further experiments are needed to determine the basis of these apparent inconsistencies.

### FGF21-Induced Acute Phosphoprotein Changes in 3T3L1 Adipocytes and White Adipose Tissues

Changes in the phosphorylation of downstream signaling molecules following FGF21 treatment in adipose/adipocytes are expected to be the most proximal effects of receptor activation, and could potentially be used as TE biomarkers in adipose tissue biopsies. We used an unbiased phosphoproteomic SILAC approach in 3T3L1 mouse adipocytes treated with FGF21 to identify such changes. As a quality control, LC-MS profiling of forward and reverse labeled samples demonstrated that the heavy isotope incorporation as well as the equal mixing of heavy and light samples were of good quality (data not shown). We subsequently identified and quantified a total of 1186 phosphopeptides corresponding to 671 unique proteins from 3T3L1 adipocytes. Among them, 137 phosphopeptides showed a greater than 1.5 fold change between vehicle and FGF21 treatment (Table S9). Of these phosphorylation events, 127 were up-regulated while 10 were down-regulated by FGF21 treatment compared to vehicle. Enriched canonical pathways from the Ingenuity Pathway Analysis tool included Insulin Receptor Signaling, IGF-1 Signaling, and Phospholipase C Signaling (see Table S10). The top 13 phosphoproteins with potential commercially available antibodies are listed in Table 3.

**Table 3:** FG21 treatment-induced changes in 3T3L1 adipocyte peptide phosphorylation.

Protein Code	Gene Symbol	Phosphopeptide Identified	Fold Change. FGF21 /Vehicle Treatment	Phospho-Antibody
Q63844	Mapk3	IADPEHDHTGFLpTEpYVATR	9.2	YES
P63085	Mapk1	VADPDHDHTGFLpTEpYVATR	7.1	YES
Q9DBR7	Ppp1r12a	RLApSTSDIEEKENR	5.2	YES
Q61686	Cbx5	RKSpSFSNSADDIK	3.0	YES
P42227	Stat3	FICVTPTTCSNTIDLPmpSPR	2.4	YES
P23804	Mdm2	(pSIS)ETEENTDELPGER	-4.7	YES
P26645	Marcks	EAAEAEPAEPSpSPAAEAEGASASSTSSPK	7.1	No, phospho site not the same
Q9Z1E4	Gys1	RSNSVDTGPSSSLSTPTEPLSP(pTS)SLGEERN	4.1	No, phospho site not the same
Q62523	Zyx	pSPGGPGPLTLK	3.8	No, phospho site not the same
Q8CGN5	Plin	LApSGGADLALGSIEK	3.6	No phospho Ab but migrates slower in SDS-PAGE when phosphorylated by PKA
Q62318	Trim28	SRpSGEGEVSGLLR	3.1	No, phospho site not the same
P46938	Yap1	SQLPTLEQDGGTPNAV(SSPGmpS)QELR	2.8	No, phospho site not the same
P81122	B230398E01Rik	SDDYmPmpSPTSVSAPK	2.5	No, phospho site not the same

Listed are the top 13 phosphopeptides showing >1.5 fold difference between FGF21 and vehicle treated 3T3L1 adipocytes. The 137 peptides, from which the 13 were derived, are listed in Table S9.

**Figure 6:**
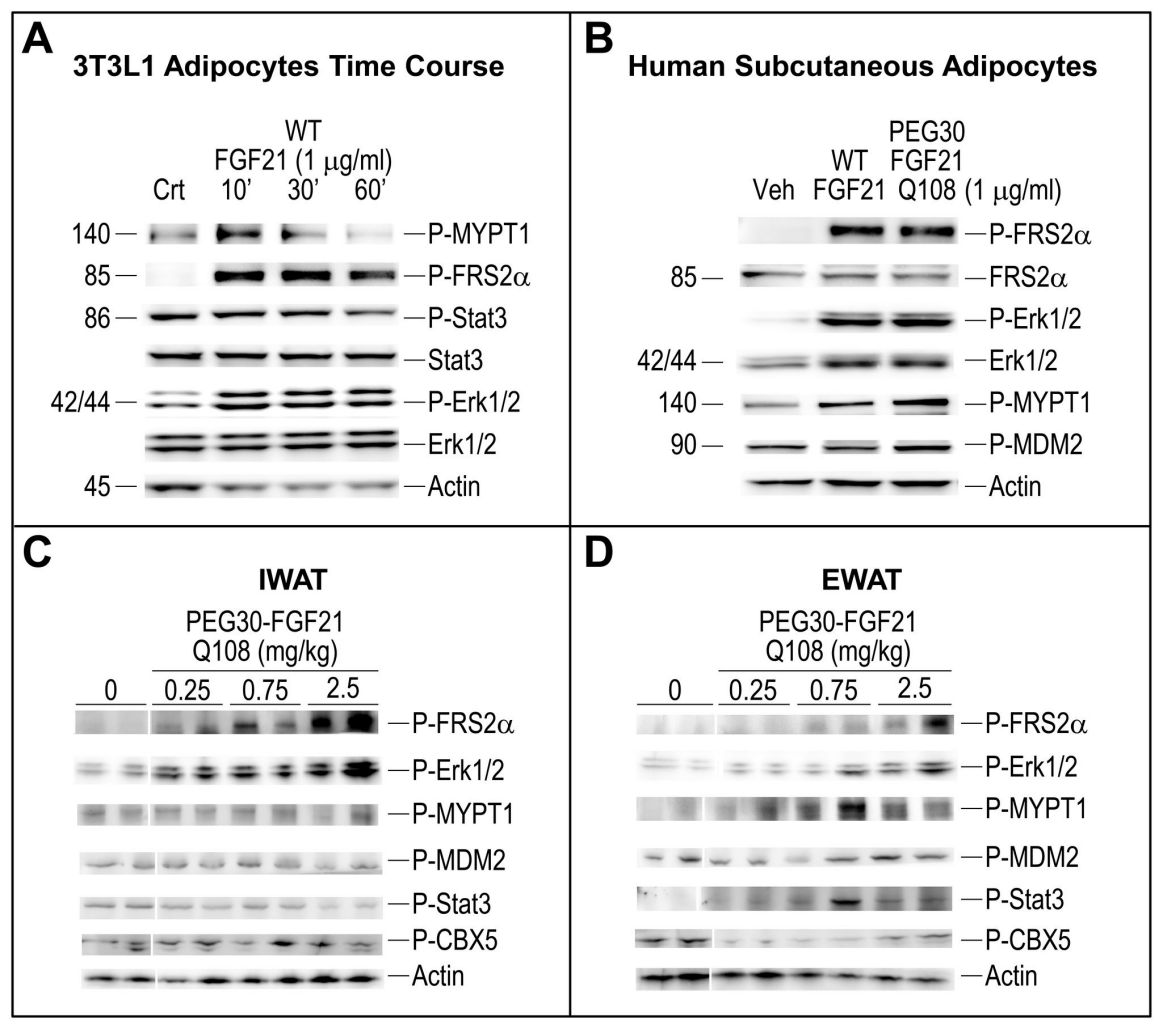
FGF21 increases phosphorylation of novel peptides in adipocytes and white adipose tissue. Analyzed extracts were from (A) mouse 3T3L1 adipocytes, (B) human subcutaneous adipocytes, and (C) IWAT or (D) EWAT tissue from C57BL/6 mice on chow diet treated for 15 min, with either vehicle (Veh), WT FGF21 or PEG30-FGF21 Q108 at the doses indicated. Shown in the Western blots are representative samples.

In addition, western blot analysis was performed on cell extracts from adipocytes and WAT extracts from the independent *in vivo* validation study at the 15 min time point using commercially available phospho-specific antibodies against 5 of the proteins predicted to increase (Frs2a, Erk1/2, Stat3, Ppp1r12a/Mypt1 and Cbx5) and one predicted to decrease (Mdm2) following FGF21 treatment. We confirmed the increase in phosphorylation of Frs2a, Erk1/2, and Ppp1r12a/Mypt1 in FGF21-treated 3T3L1 adipocytes and human adipocytes (Figure 6A,B). Interestingly, the increase in phosphorylation of the same 3 proteins and Stat3 was also seen in visceral adipose tissue (EWAT) of lean mice after FGF21 treatment *in vivo* but not in subcutaneous fat (IWAT), where increased phosphorylation of Frs2a and Erk1/2 was observed (Figure 6C,D). In addition, decreased FGF21-induced phosphorylation of Mdm2 was observed in both EWAT and IWAT while phosphorylation of Cbx5 was inconsistent in both depots. Further validation studies *in vivo* are required to establish the state of these phosphorylation events in adipose tissue following *in vivo* treatment.

## Discussion

The aim of the present study was to further interrogate the signaling events downstream of FGF21 receptor activation and, as an added bonus we identified a robust panel of target engagement (TE) biomarkers that may be useful for clinical development of FGF21-based therapeutics. For this purpose, we employed detailed transcriptomic and phosphoproteomic analyses of mouse adipose tissues and cells, respectively. Results from unbiased genome-wide studies have increased our understanding of which pathways are activated by FGF21 in this primary target tissue. Moreover, the activation of the FGFR/Klb co-receptor complex triggered phosphorylation signaling cascades that could be tied in to the gene expression changes. Finally, by monitoring a subgroup of these events in whole blood, we were able to monitor FGF21 TE either acutely or sub-chronically in vivo.

SILAC MS-based phosphoprotein enrichment and profiling identified and quantified FGF21-dependent phosphoprotein changes in 3T3L1 adipocyte cell lysates obtained 10 minutes post-treatment. Erk1 and 2 (Mapk3 and 1, respectively) were identified as two of the most robustly phosphorylated peptides in 3T3L1 adipocytes after FGF21 treatment compared to the vehicle, as expected [2]. Other phosphorylation events occurred in pathways such as the Insulin Receptor Signaling pathway (Gys1, Mapk1, Irs1, Mapk3, Rptor/4932417H02Rik, Ppp1r12a, Irs2, and Eif4ebp1 all had increased phosphorylation) and the Phospholipase C Signaling pathway (Arhgef12, Ahnak, Mapk1, Mapk3, Ppp1r12a, Arhgef2, and MARCKS all showed increased phosphorylation). Since these studies were carried out in adipocytes *in vitro*, it was important to validate these findings in adipose tissues *in vivo*. However, given that most of the phosphorylation sites we identified were novel, it was not feasible to use commercially available antibody reagents. Despite these limitations, we were able to replicate five of the FGF21-mediated phosphorylation events that were identified *in vitro*, in a visceral adipose depot (EWAT) in mice. Once additional reagents for the novel phosphorylation events become available, further validation studies will be possible.

Robust and consistent downstream transcriptional responses in white adipose tissues *in vivo* were also identified. We first selected probe sets that were consistently regulated across the three WAT depots and across the three mouse models and then sub-selected those probe sets that were robustly regulated acutely in WT mice on chow diet, resulting in 1129 and 165 probe sets, respectively (this mimics the early clinical setting of healthy normoglycemic volunteers given a single dose of recombinant FGF21).

**Figure 7:**
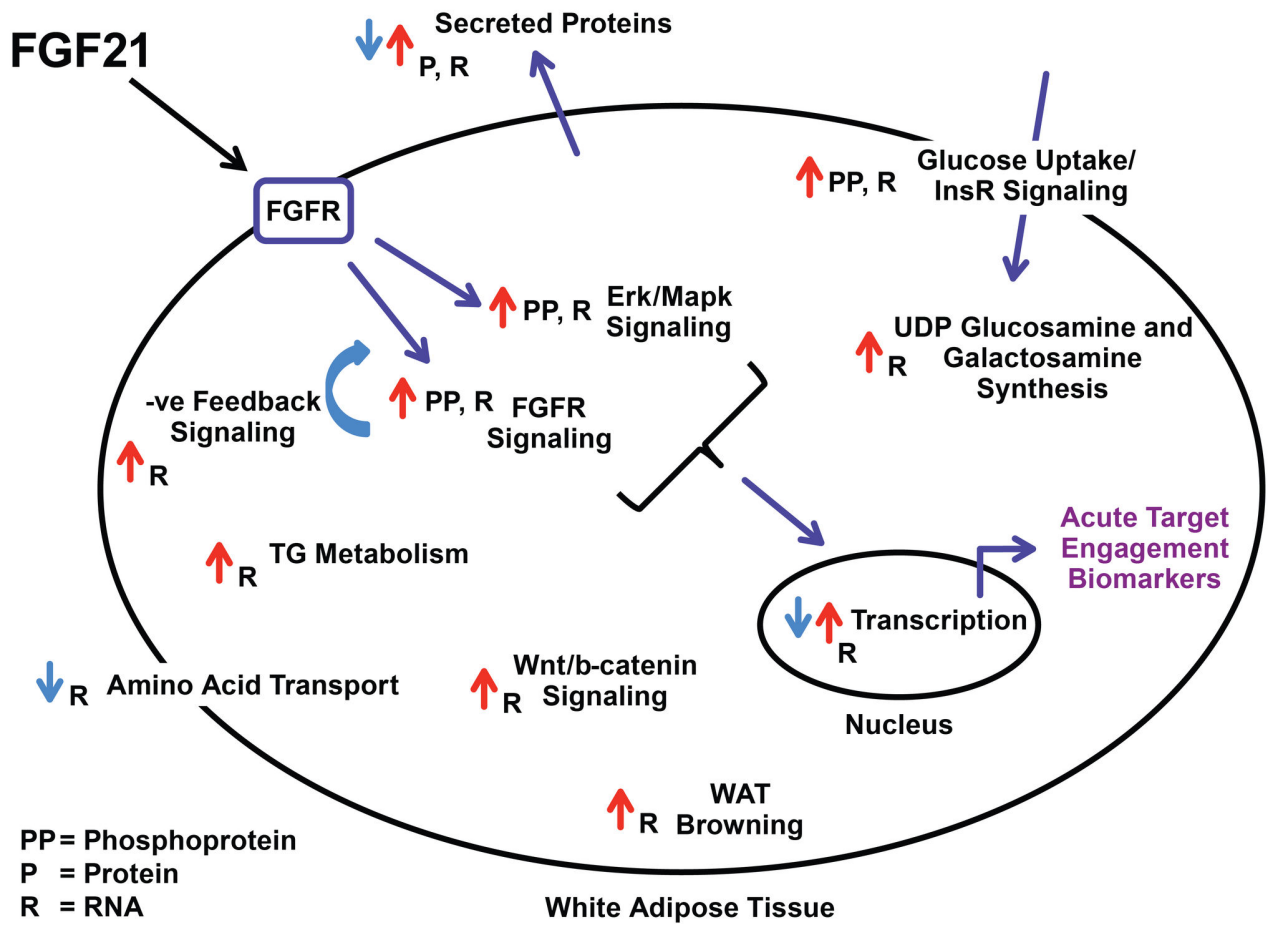
Schematic diagram of FGF21 effects in adipocytes or white adipose tissue.

Pathway and GO term enrichment analysis on the broader gene set (1129 probe sets) identified metabolic pathways known to be affected by FGF21 as well as pathways not previously associated with this protein. Multiple metabolic and signaling pathways were enriched, such as Fgfr, Erk/Mapk, Pi3k/Akt, Igf-1, and mTor signaling, triglyceride synthesis and degradation, glucose uptake, amino acid transport and energy expenditure (see Table S4 and the summary schematic diagram in Figure 7). Perhaps not surprisingly, the most robustly regulated genes were those involved in negative feedback regulation of Fgfr signaling (Dusp4 and Spry4), even at the lowest doses of FGF21 investigated.

Depicted are phosphorylation (PP), protein (P), or RNA (R) changes after FGF21 treatment in vitro (3T3L1 adipocytes) or in vivo (WAT depots). See Table S2, S3, S4, S9 for details.Most genes regulated by FGF21 identified by our studies have unknown biological significance in terms of beneficial consequences of FGF21 activation, but can nevertheless be used as robust TE biomarkers. Others have known functions and their regulation by FGF21 either supports (increased expression of Ucp1 and energy expenditure [20]) or seems to contradict (decreased expression of Sfrp5 and a worsening of metabolic dysfunction [33]) a beneficial role of FGF21. Indeed, Sfrp5 expression was consistently down-regulated by FGF21 across the three WAT depots and across the three mouse models used. In addition, Sfrp5 expression was higher in WAT depots from db/db mice when compared to WT mice suggesting a “reversal” of the disease phenotype by FGF21 treatment (Figure S3). This is in contrast to the reported decreased expression in WAT from ob/ob mice [33]. Furthermore, changes in Sfrp5 expression following FGF21 treatment in WAT approached the level of expression of Sfrp5 in BAT under basal conditions, indicative of a white adipose tissue “browning” effect by FGF21 (Figure S4). There are also conflicting reports on the role of Sfrp5 in human adipose biology [34,35], thus the biological impact of a down-regulation of this gene by FGF21 warrants further investigation.

FGF21 expression has been shown to be up-regulated by PPARγ agonist treatment in adipose tissue and adipocytes [8,9,36,37], and there is also evidence that FGF21 treatment in turn regulates PPARγ activity [9]. We compared the transcriptional responses in db/db EWAT following FGF21 treatment in the current study to that following rosiglitazone (PPARμ agonist) treatment in a previously published study [8]. Of the genes regulated by both treatments, the majority was regulated in the same direction (for example Ucp1 and Hsd17b12 were both up-regulated (see Figure S6 and Table S11). Whether this overlap reflects an overall beneficial metabolic outcome in common to both treatments or more direct effects remain to be elucidated.

Several of the transcripts that we identified to be regulated by FGF21 treatment, encode secreted proteins. From a biomarker perspective, these would allow for the development of blood-based protein detection assays to measure FGF21 target engagement, which would be less invasive in a clinical setting than adipose tissue biopsies (for RNA and phosphoprotein biomarkers). Over 300 such transcripts were identified following acute treatment with FGF21. The plasma levels of 5 of these proteins for which there were commercially available antibodies were determined in a validation study and the FGF21-induced changes in plasma agreed with their gene expression changes in IWAT for at least 2 of them (Ccl11 and Il1rn). Three other proteins were tested in the circulation but they did not show dose-dependent regulation using the available reagents, although there was a change in plasma protein levels for all three proteins at the highest dose of FGF21, in agreement with the RNA profiling data.

Further studies are required to validate the additional potential secreted proteins once reagents become available. However, one secreted protein of relevance to these studies is adiponectin, which has recently been described as having a key role in mediating FGF21’s beneficial metabolic effects in mice [17,18]. In the present study, the mRNA of this gene in WAT was not altered following FGF21 treatment, perhaps reflecting the high basal level of expression in this tissue. Consistent with this observation, we also could not detect an increase in secreted adiponectin in either the culture medium of 3T3L1 adipocytes or in the circulation of mice treated in vivo (Figure S5). In contrast, we did recapitulate published data with rosiglitazone in parallel experiments, where secreted adiponectin levels were significantly increased. Moreover, the absence of increases in adiponectin mRNA and protein levels could not be attributed to a lack of FGF21 effect, since we observed a robust increase in Erk phosphorylation, a proximal TE biomarker, both *in vitro* and *in vivo* (Figure S5). Further studies are required to determine whether these differences reflect nuanced experimental conditions such as experimental design, cell / mouse sources or reagent sources.

In the current series of experiments, the impact of FGF21 on BAT gene expression was more robust than in the WAT; while this is consistent with the increased oxidation of glucose observed in db/db mice following PEG30-FGF21 treatment [5], further experiments are needed to fully elucidate any tissue-specific differences between the metabolic effects of FGF21 on BAT and WAT.

In summary, transcriptomic and phosphoproteomic profiling analyses have been performed in mouse adipose tissues *in vivo* and 3T3L1 adipocytes, respectively, following acute FGF21 treatment to further elucidate the downstream pathways affected. In addition to confirming a number of previously reported signaling cascades, our studies also uncovered several novel transcriptional and post-translational (phosphorylation) events that warrant further investigation. Several key biomarkers were identified that may serve not only as clinical readouts of FGF21 target engagement but might also help elucidate the mechanisms of action in this primary target tissue that mediate FGF21’s beneficial metabolic effects. Specifically, we identified distinct transcriptional effects of FGF21 in brown and white adipose tissue. This opens up a window for further studies to investigate both the therapeutic benefits of FGF21 and to compare the metabolic differences between these distinct adipose tissue types.

## Supporting Information

Figure S1 **FGF21 treatment-induced transcriptional signatures in mouse adipose tissues.** The signature counts are plotted for each adipose depot across the three mouse models and for both time points (2 and 5 day). Probe sets that were regulated at least 1.2 fold and had a 1-way ANOVA p<0.05 between PEG30-FGF21 Q108 (2.5 mg/kg) and vehicle treatments were included in the signature.
(PDF)

Figure S2 **Acute FGF21 treatment-induced RNA markers in white adipose tissues.** Clustergrams for the top 32 RNA markers (Table 1) from EWAT (A) and RPWAT (B) are represented (see Figure 2 for IWAT). Plotted are the logRatio values on a scale of +/- 0.6 (+/- 4 fold) with magenta and cyan signifying up- and down-regulated genes, respectively. Each row is the average of up to 5 animals for that treatment group. Gene names are shown below the clustergram. Native = WT FGF21; PEG-L and PEG-H = PEG30-FGF21 Q108 at 0.75 and 2.5 mg/kg, respectively; and 2d and 5d correspond to number of days of treatment.
(PDF)

Figure S3 **Sfrp5 expression is decreased in WAT by FGF21 treatment and is a reversal of the “disease” phenotype.** Plotted in the bar graph are the fold change values for Sfrp5 (probe set: merck-NM_018780_at, see Table S1) across the three WAT depots and the three mouse models. The two “disease” phenotypes shown are the WT HFD/WT Chow and the db/db Chow/WT Chow comparisons. Red bars reach 1-way ANOVA p<0.05.
(PDF)

Figure S4 **Acute FGF21 treatment-induced RNA markers in white adipose tissues.** Plotted in the box plots are the logIntensity values for the top 32 RNA markers identified in WAT following acute FGF21 treatment (Figure 2 and Table 1). The data plotted is from IWAT and BAT of WT-chow fed mice after either vehicle or PEG30-FGF21 Q108 (2.5 mg/kg) treatment (at both time points, 2 and 5 days). Ucp1 (AF) was added as a white adipose “browning” control.
(PDF)

Figure S5 **Adiponectin secretion is not affected by FGF21 treatment of 3T3L1 adipocytes or in mice in vivo.** Shown are Adiponectin protein levels in either (A) media collected from 3T3L1 adipocytes treated for 24 with either vehicle, WT FGF21 (Biovendor LLC, Asheville, NC) or Rosiglitazone at the doses indicated, or (B) plasma from C57BL/6 mice treated for 1 or 6 hours with either vehicle or WT FGF21 at 1 mg/kg (Phoenix Pharmaceutical, Burlingame, CA). Media and plasma adiponectin levels were evaluated by ELISA kit according to the manufacturer’s instructions (MSD, Rockville, MD). Percentage phosphorylated Erk1/2 is also shown below the Adiponectin graphs as control for FGF21 receptor activation. Samples for Erk1/2 evaluation were obtained 15 minutes post treatment in both cases. Differentiated 3T3L1 adipocytes were serum depleted 2 hours in 0.5% fatty acid free BSA before treatment, while 20-week old C57Bl6 mice were fasted for 2h prior to treatment (i.p.) followed by euthanasia by CO2 asphyxiation 1 and 6 hours post treatment.
(PDF)

Figure S6 **Overlap between FGF21 and PPARγ agonist-induced transcriptional effects in EWAT from db/db mice.** Plotted are the genes that were previously reported to be regulated by PPARγ agonists in EWAT from db/db mice [8] (379 genes represented by 452 probes), and that were in common with the 1129 FGF21-responsive probe sets in WAT identified in this report (see Table S11). The numbers indicate the number of data points (probeset-to-probe combinations) in each quandrant.
(PDF)

Table S1 **FGF21 treatment-induced consistent gene expression changes in mouse adipose tissues.** Listed are the 1129 probe sets that had an N-way ANOVA p<0.001 in all three WAT depots (intersection). The N-way ANOVA was performed by incorporating all FGF21 treatments for 2 days and 5 days and mouse strains/disease models. The fold change and 1-way ANOVA p values for both the acute (2 day) and sub-chronic (5 day) treatments of PEG30-FGF21 Q108 (2.5 mg/kg) in IWAT, EWAT and RPWAT in all three mouse models (WT-Chow, WT-HFD, and db/db-Chow) are shown as is the data from the validation study (qPCR). The two “disease” comparisons are also included (WT-HFD/ WT-Chow, and db/db-Chow/WT-Chow).
(XLSX)

Table S2 **GO term enrichment analysis of the FGF21 treatment-induced signature in WAT.** Listed are the GO terms with expectation p value <0.1 for enrichment in the 1129 gene set (Table S1). The probe set IDs associated with each GO term are shown in column J.
(XLSX)

Table S3 **Canonical pathway enrichment analysis, from the Ingenuity Pathway Analysis tool, of the FGF21 treatment-induced signature in WAT.** Listed are the canonical pathways enriched in the 1129 gene set (Table S1). The gene symbols associated with each canonical pathway are shown in column E.
(XLSX)

Table S4 **Canonical pathway enrichment analysis, from the Ingenuity Pathway Analysis tool, of the FGF21 treatment-induced signature in WAT.** Listed is a subset of canonical pathways enriched in the 1129 gene set (Table S3) together with the gene expression data from the current study as described in the legend of Table S1.
(XLSX)

Table S5 **FGF21 treatment induces robust and consistent gene expression changes in brown adipose tissue.** Listed are the 1634 probe sets regulated by a single 2.5mg/kg dose of PEG-30-FGF21 Q108 in WT mice on chow diet. The data for both acute (2 day) and sub-chronic (5 day) dosing are shown.
(XLSX)

Table S6 **Canonical pathway enrichment analysis, from the Ingenuity Pathway Analysis tool, of the FGF21 treatment-induced signature in BAT.** Listed are the canonical pathways enriched in the 1634 gene set (Table S5). The gene symbols associated with each canonical pathway are shown in column E.
(XLSX)

Table S7 **FGF21 treatment-induced gene expression changes of transcripts encoding potential secreted proteins.** Listed are the 334 potential secreted proteins that were regulated by a single 2.5 mg/kg dose of PEG30-FGF21 Q108 (1.2 fold and p<0.05) in at least one of three white adipose tissues (IWAT, EWAT, RPWAT). The gene expression data from the current study is included as described in the legend of Table S1.
(XLSX)

Table S8 **Plasma levels of secreted proteins identified in WAT by Affymetrix microarray profiling.** C57BL/6 mice on chow diet were treated with a single dose of vehicle or PEG30-FGF21 Q108 at one of three doses (0.25, 0.75, or 2.5 mg/kg). Plasma Ccl11, Cxcl2, Igf1, Il1rn, and Kitl levels were measured 24 hours and 48 hours post dose. Listed are the mean values for each group as plotted in Figure 5.
(XLSX)

Table S9 **FG21-induced changes in 3T3L1 adipocyte peptide phosphorylation.** Listed are the top 137 phosphopeptides identified by either data dependent or targeted MS/MS acquisition from manually validated features showing >1.5 fold difference between FGF21 and vehicle treated 3T3L1 adipocytes.
(XLSX)

Table S10 **Canonical pathway enrichment analysis, from the Ingenuity Pathway Analysis tool, of the FGF21 treatment-induced changes in protein phosphorylation in 3T3L1 adipocytes.** Listed are the canonical pathways enriched in the 137 protein set (Table S9). The gene symbols associated with each canonical pathway are shown in column E.
(XLSX)

Table S11 **Overlap of the FGF21 and PPARγ agonist treatment responses in EWAT from db/db mice.** Listed are the genes that were previously reported to be regulated by PPARγ agonists in EWAT from db/db mice [8], (379 genes represented by 452 probes), and that were in common with the 1129 FGF21-responsive probe sets in WAT identified in this report (see Table S1). There were 83 resulting probeset-to-probe combinations (since both studies were performed on different array platforms). The following data (fold change and p value) are included: 1) the 2.5 mg/kg dose of PEG30-FGF21 Q108 at the 5 day time point in db/db-Chow (current study), 2) the db/db-Chow vs WT-Chow “disease” comparison (current study), 3) the Rosiglitazone and COOH treatments at the 8 day time point (previously published study), and 4) the db/db-Chow vs WT-Chow “disease” comparison (previously published study). See Figure S6.
(XLSX)
