# Supplementary figures and images for: Downstream Signaling Pathways in Mouse Adipose Tissues Following Acute In Vivo Administration of Fibroblast Growth Factor 21
Source: PLoS One. 2013 Sep 6;8(9):e73011. doi: 10.1371/journal.pone.0073011 (PMC3765203; doi:10.1371/journal.pone.0073011)

Supplemental Figure S1

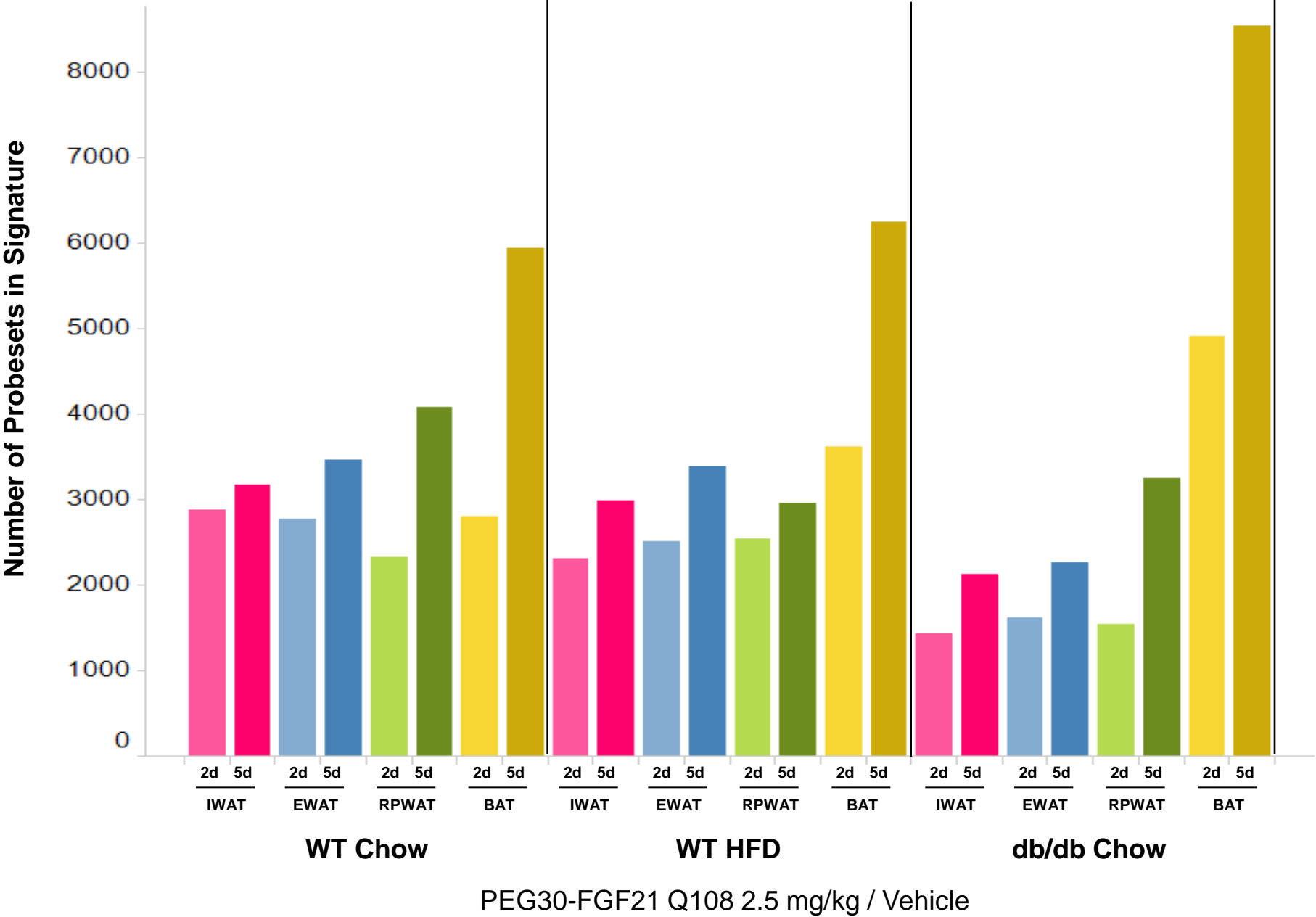

Supplement: Figure S1 — FGF21 treatment-induced transcriptional signatures in mouse adipose tissues. The signature counts are plotted for each adipose depot across the three mouse models and for both time points (2 and 5 day). Probe sets that were regulated at least 1.2 fold and had a 1-way ANOVA p<0.05 between PEG30-FGF21 Q108 (2.5 mg/kg) and vehicle treatments were included in the signature. (PDF) [file pone.0073011.s001.pdf]

Supplemental Figure S2

A

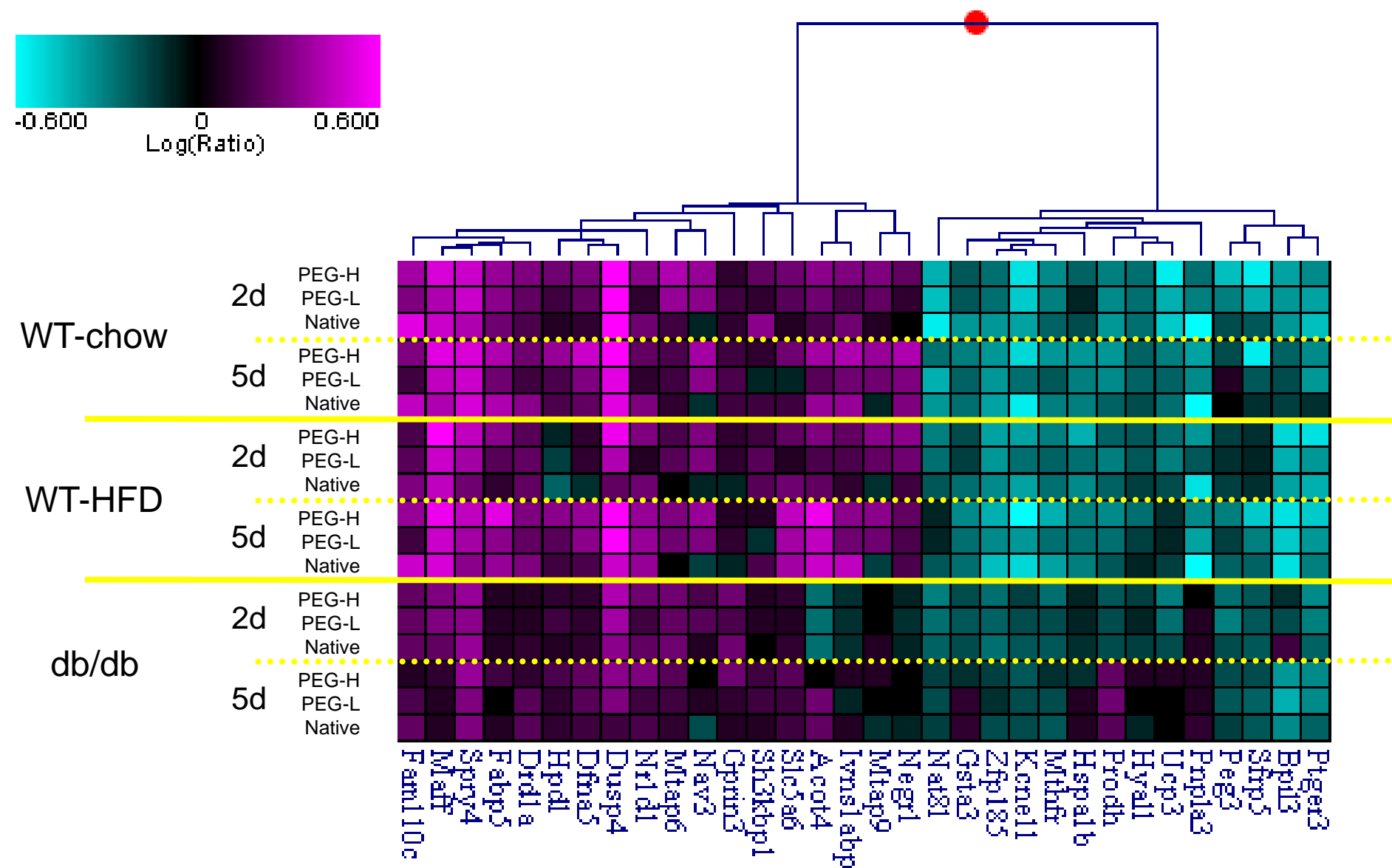

Supplemental Figure S2

B

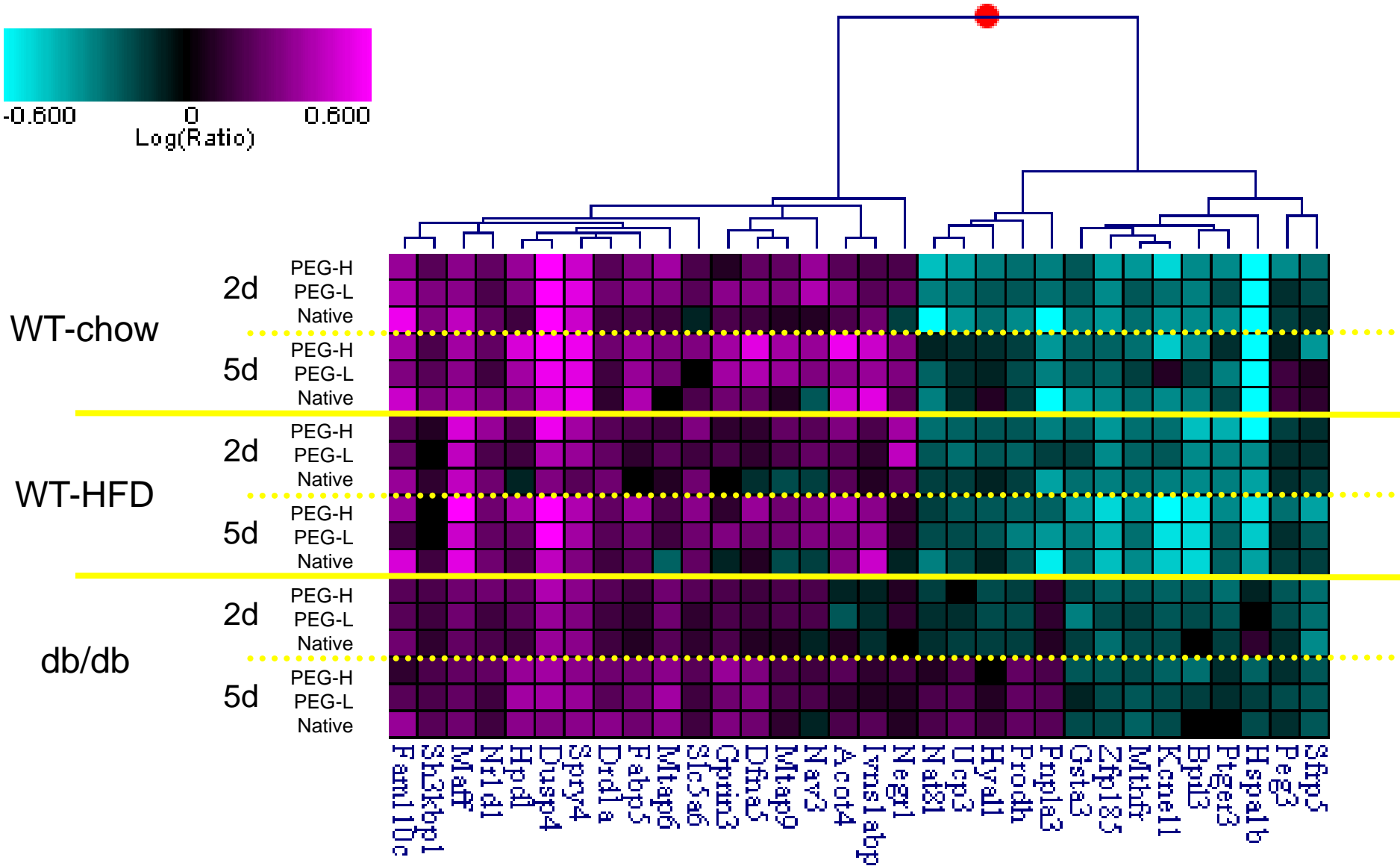

Supplement: Figure S2 — Acute FGF21 treatment-induced RNA markers in white adipose tissues. Clustergrams for the top 32 RNA markers (Table 1) from EWAT (A) and RPWAT (B) are represented (see Figure 2 for IWAT). Plotted are the logRatio values on a scale of +/- 0.6 (+/- 4 fold) with magenta and cyan signifying up- and down-regulated genes, respectively. Each row is the average of up to 5 animals for that treatment group. Gene names are shown below the clustergram. Native = WT FGF21; PEG-L and PEG-H = PEG30-FGF21 Q108 at 0.75 and 2.5 mg/kg, respectively; and 2d and 5d correspond to number of days of treatment. (PDF) [file pone.0073011.s002.pdf]

Supplemental Figure S5

**A 3T3L1 Adipocytes**

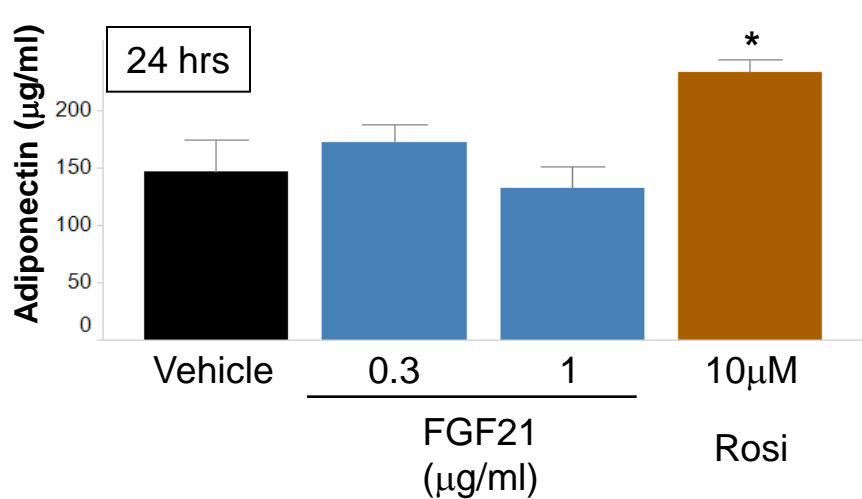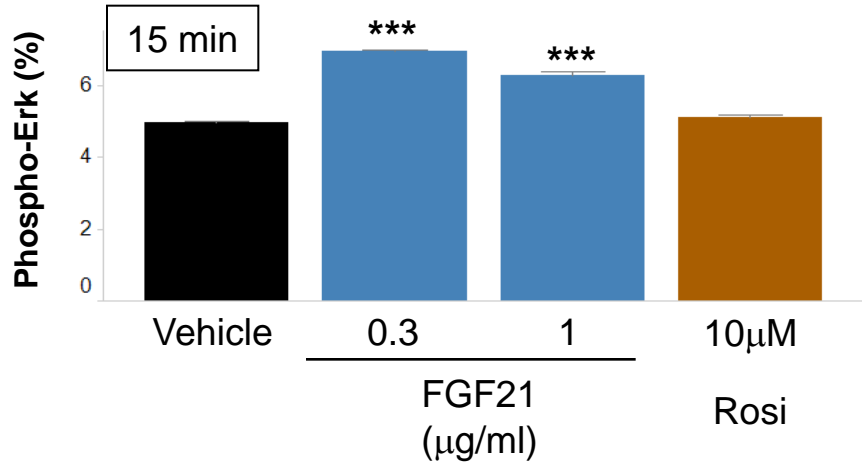

**B Mouse In vivo**

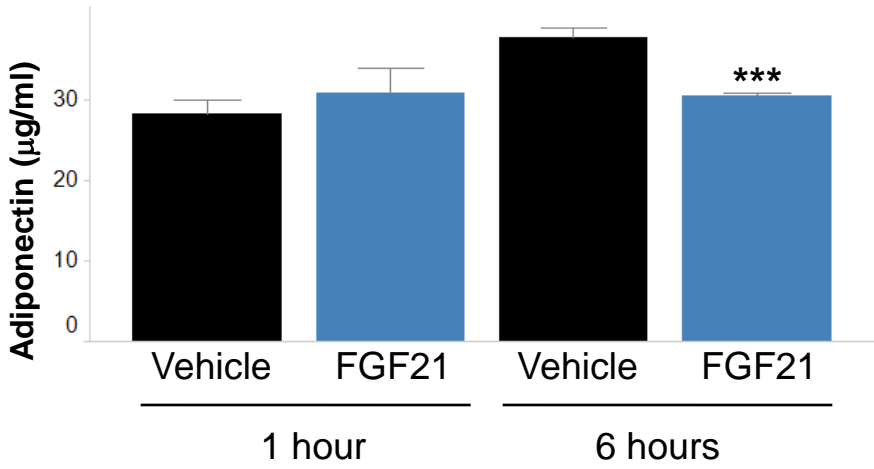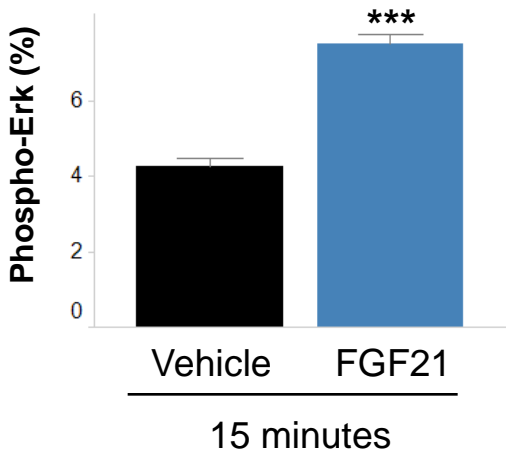

Supplement: Figure S5 — Adiponectin secretion is not affected by FGF21 treatment of 3T3L1 adipocytes or in mice in vivo. Shown are Adiponectin protein levels in either (A) media collected from 3T3L1 adipocytes treated for 24 with either vehicle, WT FGF21 (Biovendor LLC, Asheville, NC) or Rosiglitazone at the doses indicated, or (B) plasma from C57BL/6 mice treated for 1 or 6 hours with either vehicle or WT FGF21 at 1 mg/kg (Phoenix Pharmaceutical, Burlingame, CA). Media and plasma adiponectin levels were evaluated by ELISA kit according to the manufacturer’s instructions (MSD, Rockville, MD). Percentage phosphorylated Erk1/2 is also shown below the Adiponectin graphs as control for FGF21 receptor activation. Samples for Erk1/2 evaluation were obtained 15 minutes post treatment in both cases. Differentiated 3T3L1 adipocytes were serum depleted 2 hours in 0.5% fatty acid free BSA before treatment, while 20-week old C57Bl6 mice were fasted for 2h prior to treatment (i.p.) followed by euthanasia by CO2 asphyxiation 1 and 6 hours post treatment. (PDF) [file pone.0073011.s005.pdf]

Supplemental Figure S6

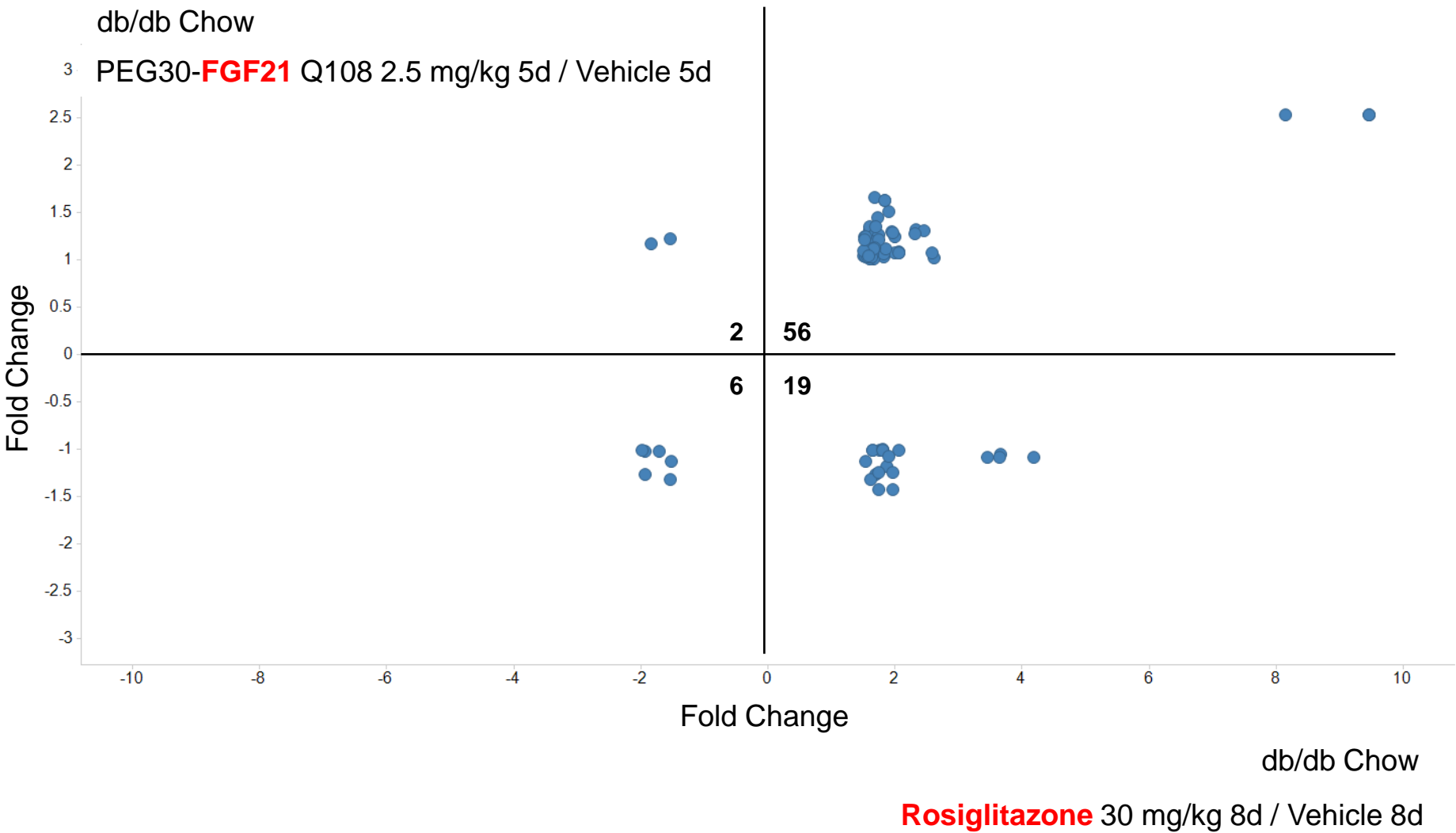

Supplement: Figure S6 — Overlap between FGF21 and PPARγ agonist-induced transcriptional effects in EWAT from db/db mice. Plotted are the genes that were previously reported to be regulated by PPARγ agonists in EWAT from db/db mice [8] (379 genes represented by 452 probes), and that were in common with the 1129 FGF21-responsive probe sets in WAT identified in this report (see Table S11). The numbers indicate the number of data points (probeset-to-probe combinations) in each quandrant. (PDF) [file pone.0073011.s006.pdf]
