# Supplementary material for: Downstream Signaling Pathways in Mouse Adipose Tissues Following Acute In Vivo Administration of Fibroblast Growth Factor 21
Source: PLoS One. 2013 Sep 6;8(9):e73011. doi: 10.1371/journal.pone.0073011 (PMC3765203; doi:10.1371/journal.pone.0073011)

Supplemental Figure S3

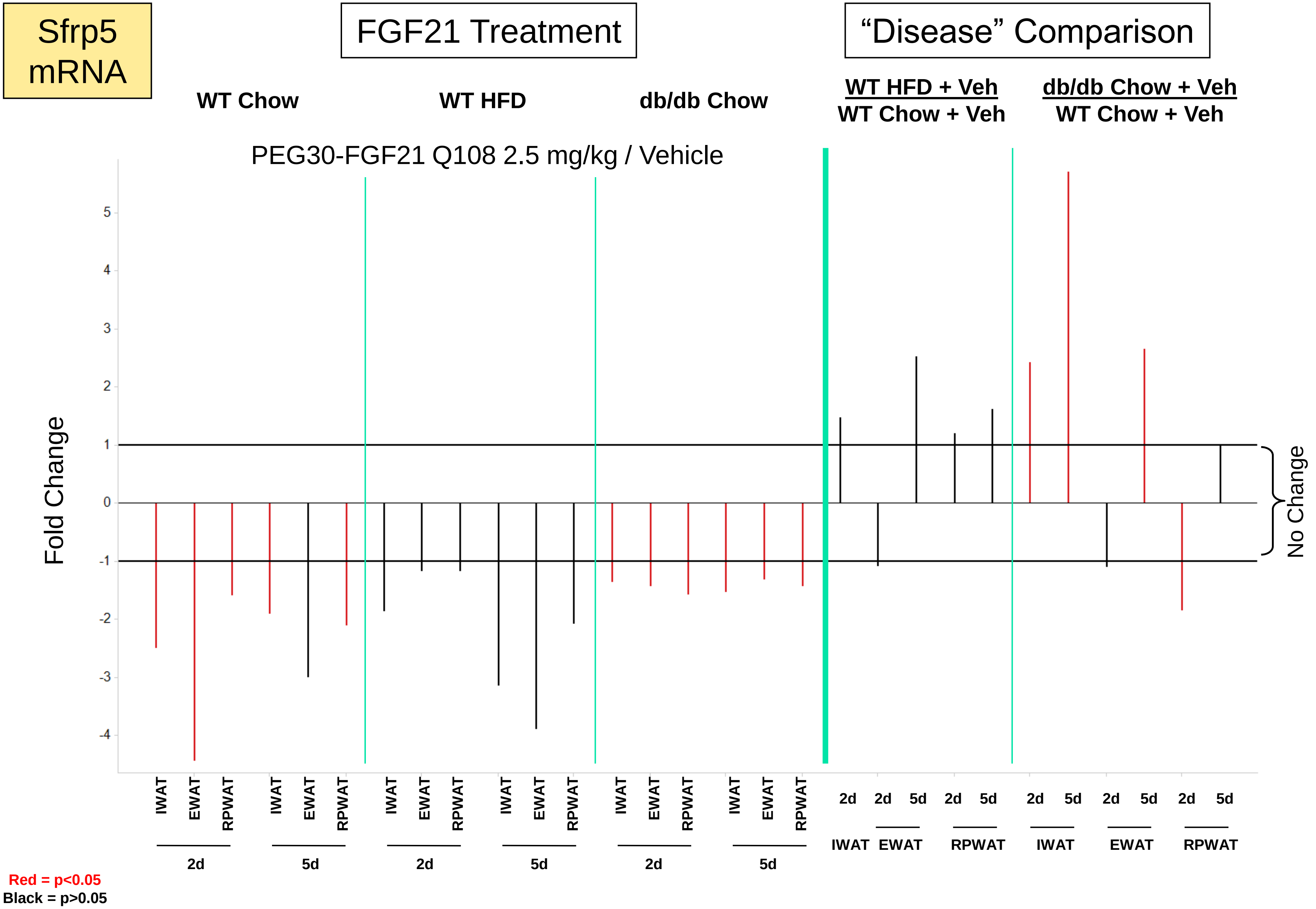

Supplement: Figure S3 — Sfrp5 expression is decreased in WAT by FGF21 treatment and is a reversal of the “disease” phenotype. Plotted in the bar graph are the fold change values for Sfrp5 (probe set: merck-NM_018780_at, see Table S1) across the three WAT depots and the three mouse models. The two “disease” phenotypes shown are the WT HFD/WT Chow and the db/db Chow/WT Chow comparisons. Red bars reach 1-way ANOVA p<0.05. (PDF) [file pone.0073011.s003.pdf]
