# Supplementary material for: Downstream Signaling Pathways in Mouse Adipose Tissues Following Acute In Vivo Administration of Fibroblast Growth Factor 21
Source: PLoS One. 2013 Sep 6;8(9):e73011. doi: 10.1371/journal.pone.0073011 (PMC3765203; doi:10.1371/journal.pone.0073011)

Supplemental Figure S4

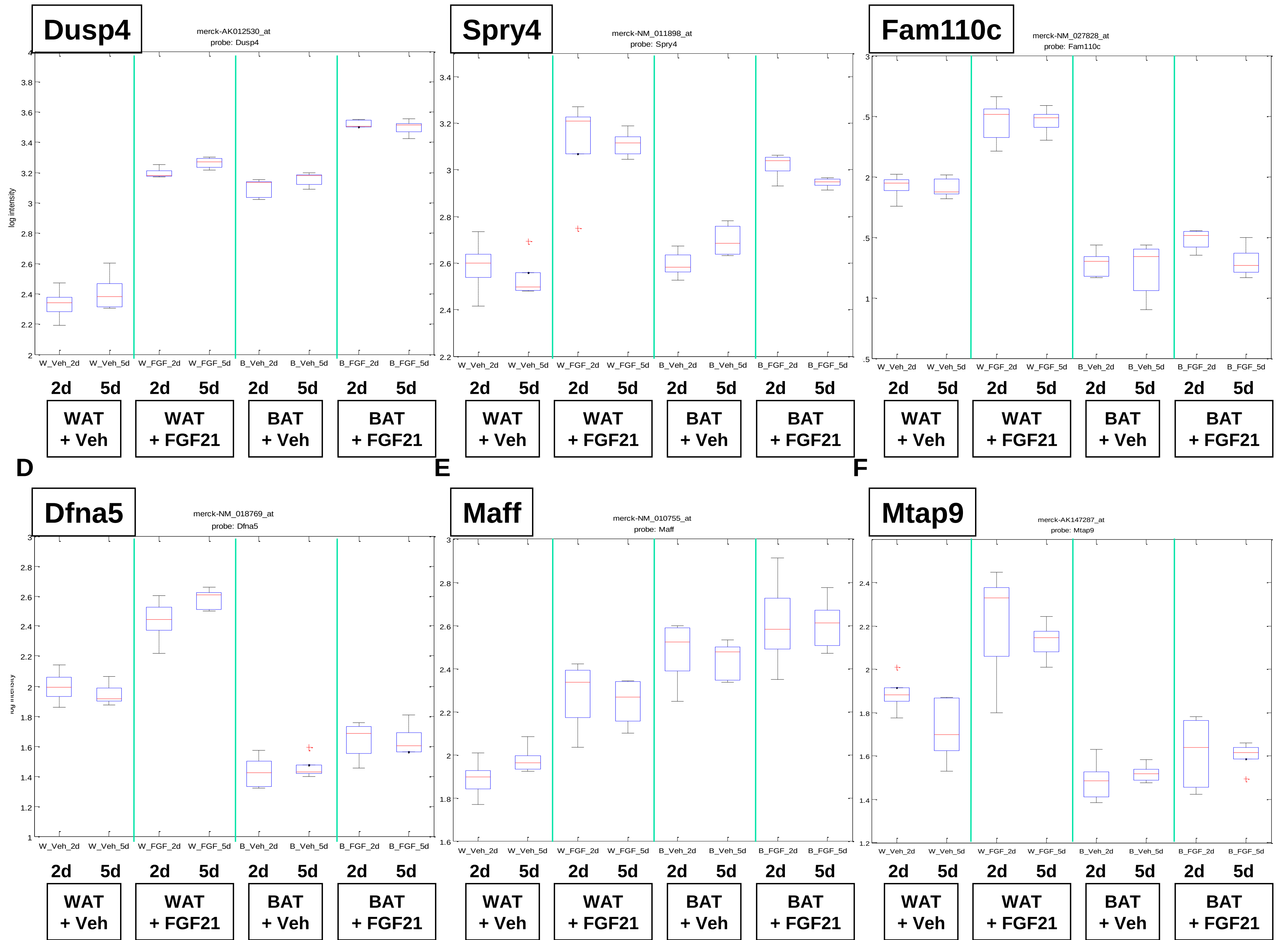

Supplemental Figure S4

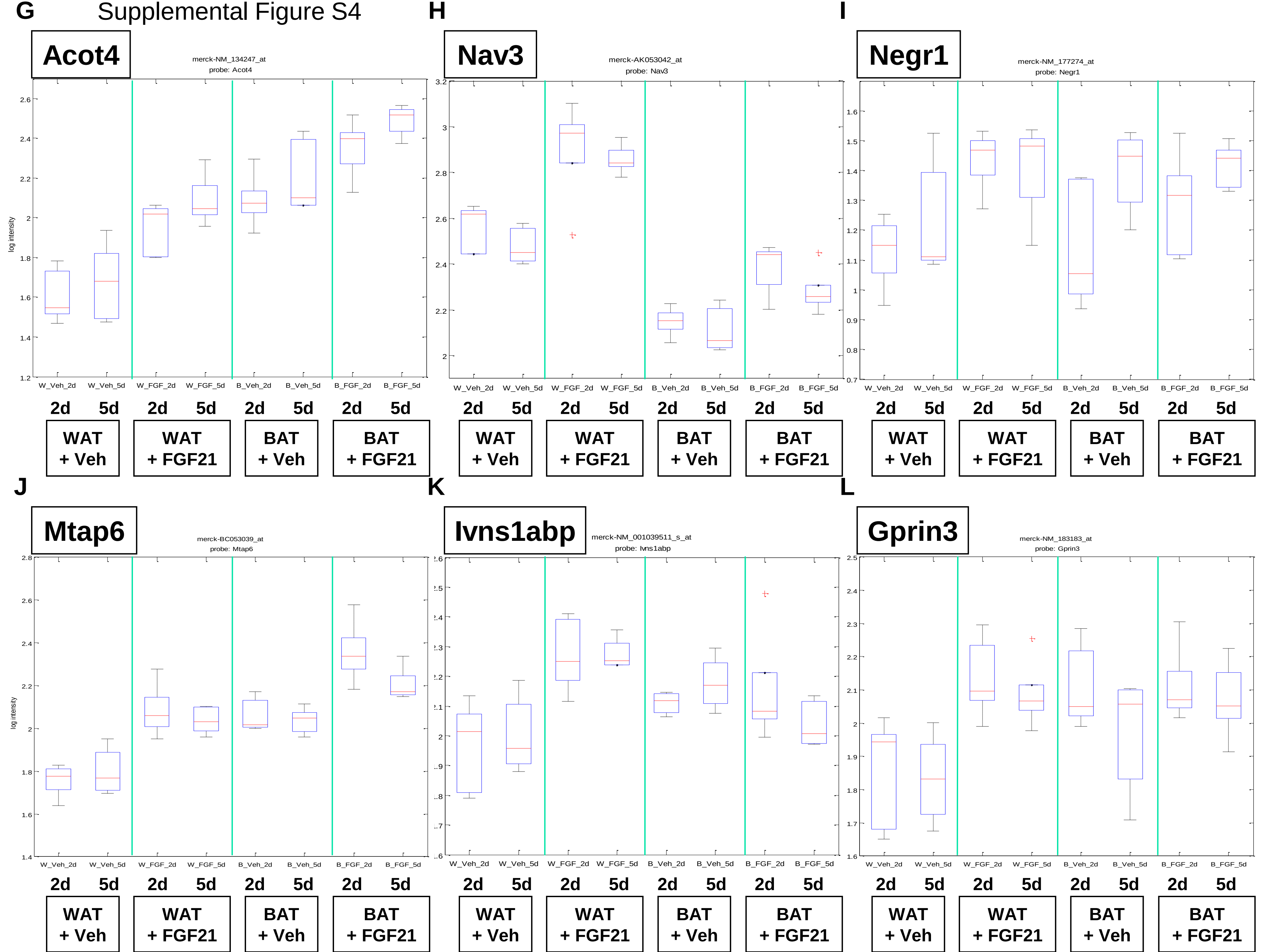

Supplemental Figure S4

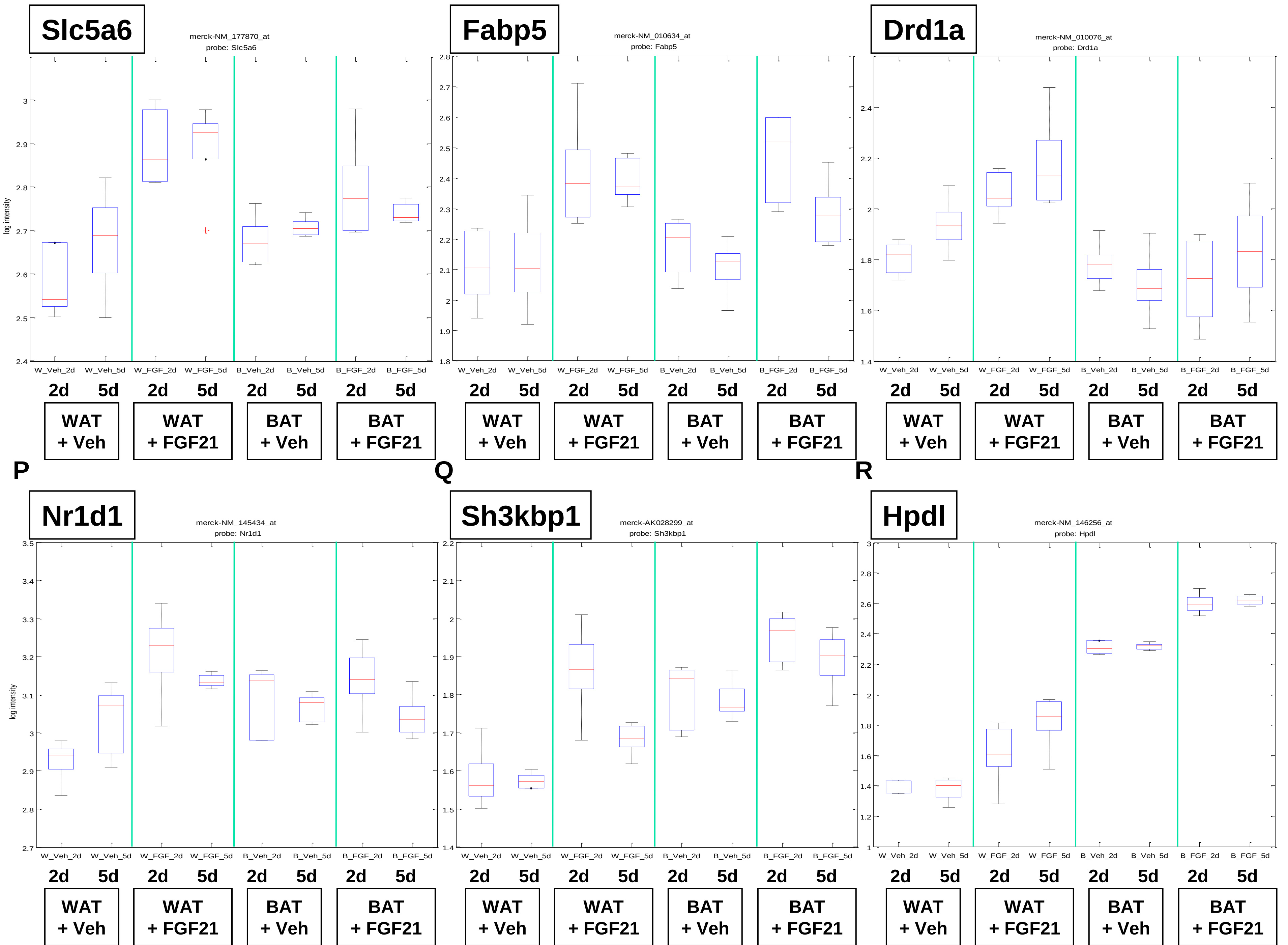

Supplemental Figure S4

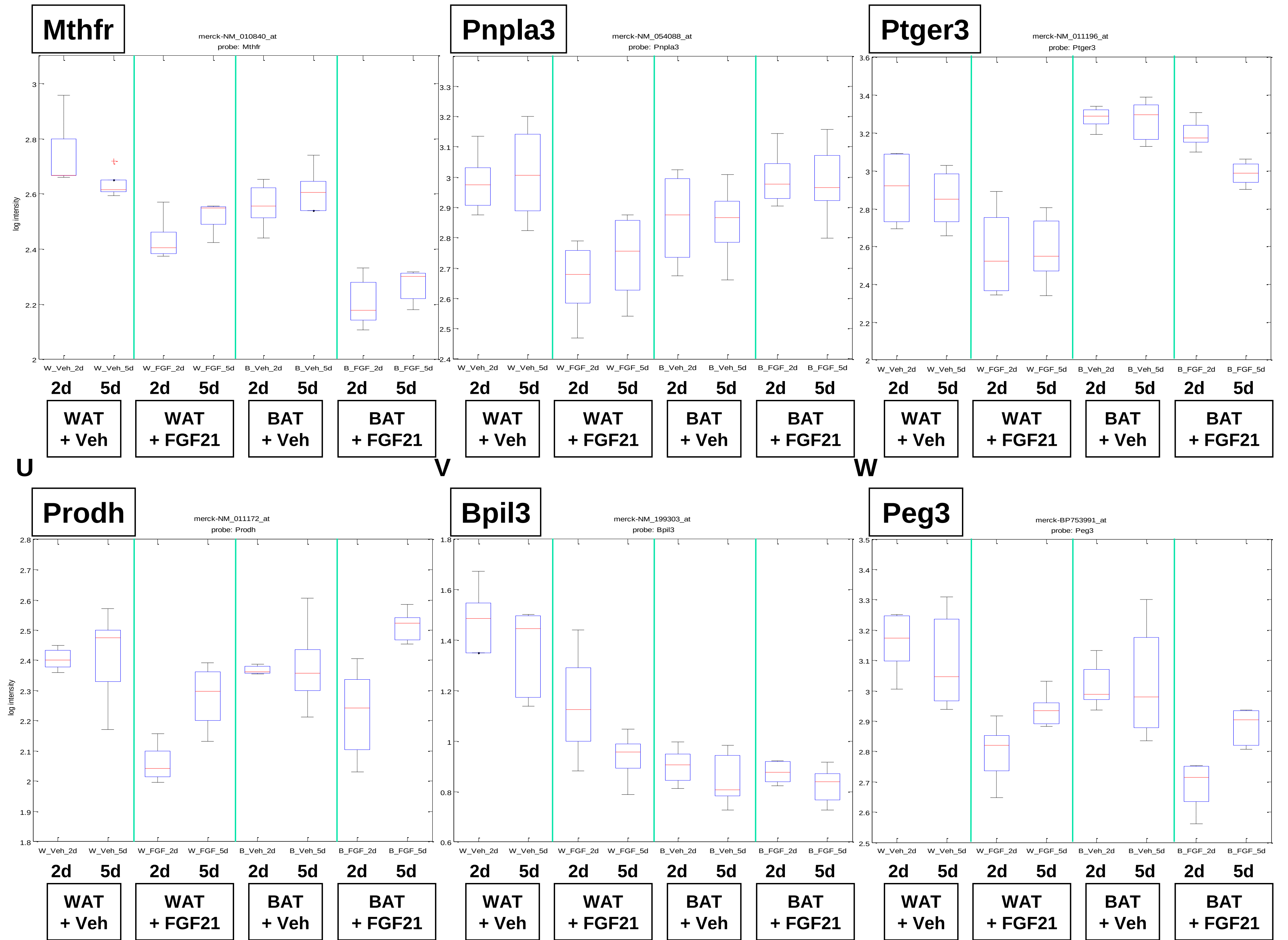

Supplemental Figure S4

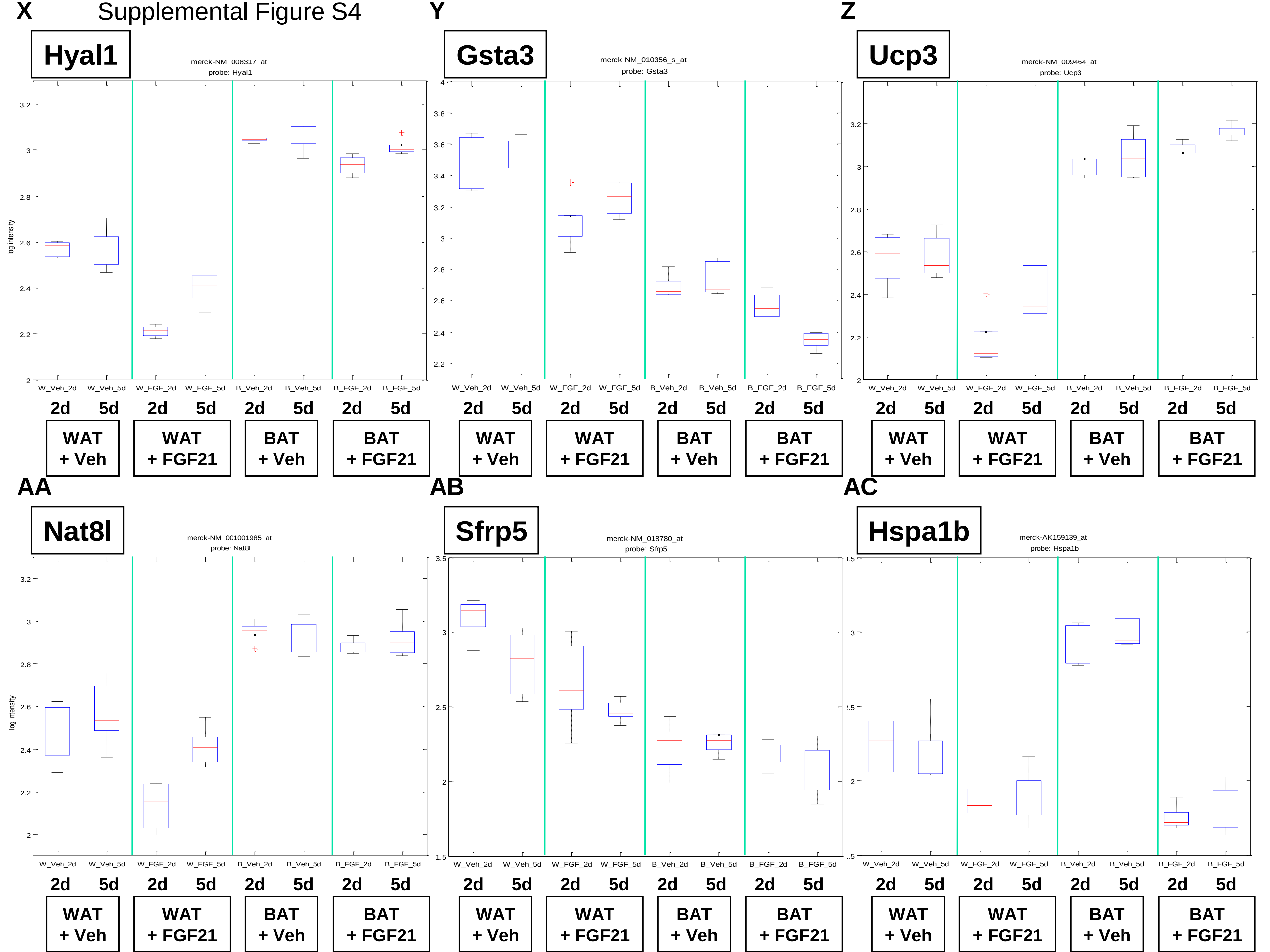

Supplemental Figure S4

AD AE AF

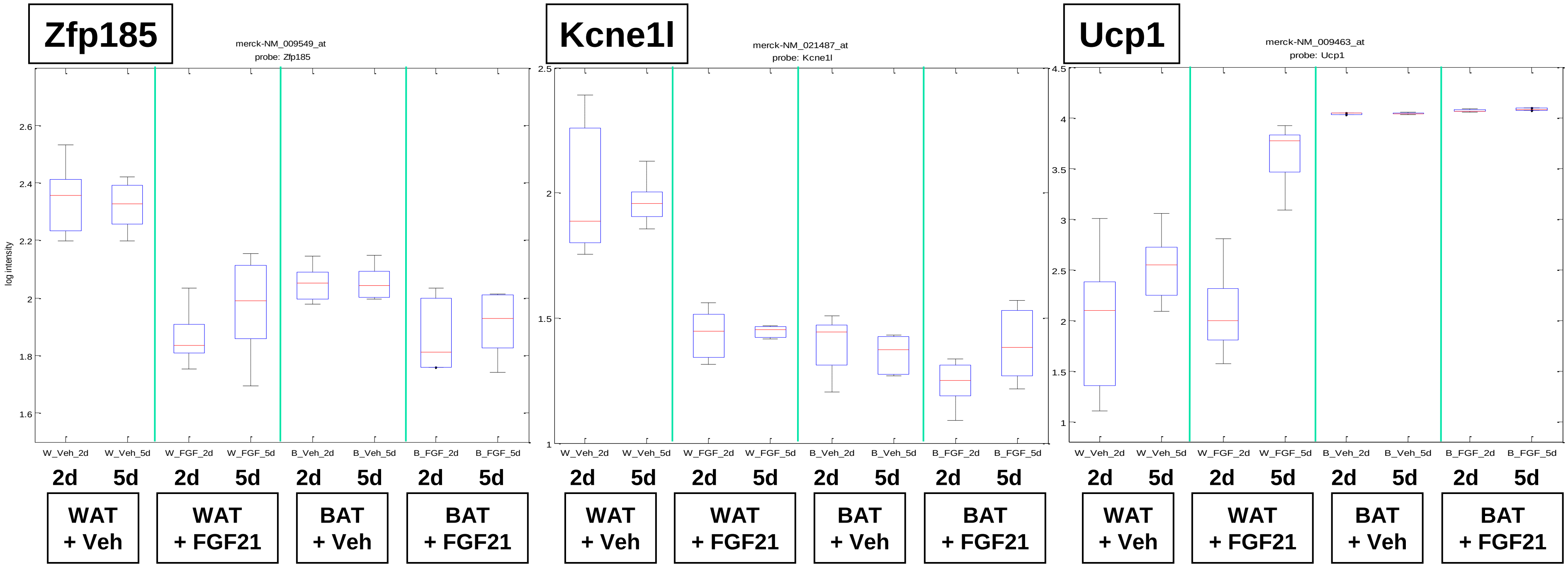

Supplement: Figure S4 — Acute FGF21 treatment-induced RNA markers in white adipose tissues. Plotted in the box plots are the logIntensity values for the top 32 RNA markers identified in WAT following acute FGF21 treatment (Figure 2 and Table 1). The data plotted is from IWAT and BAT of WT-chow fed mice after either vehicle or PEG30-FGF21 Q108 (2.5 mg/kg) treatment (at both time points, 2 and 5 days). Ucp1 (AF) was added as a white adipose “browning” control. (PDF) [file pone.0073011.s004.pdf]
